# Therapeutic Potential of α- and β-Pinene: A Miracle Gift of Nature

**DOI:** 10.3390/biom9110738

**Published:** 2019-11-14

**Authors:** Bahare Salehi, Shashi Upadhyay, Ilkay Erdogan Orhan, Arun Kumar Jugran, Sumali L.D. Jayaweera, Daniel A. Dias, Farukh Sharopov, Yasaman Taheri, Natália Martins, Navid Baghalpour, William C. Cho, Javad Sharifi-Rad

**Affiliations:** 1 Student Research Committee, School of Medicine, Bam University of Medical Sciences, Bam 44340847, Iran; bahar.salehi007@gmail.com; 2 G. B. Pant National Institute of Himalayan Environment and Sustainable Development, Kosi-Katarmal, Almora-263643, Uttarakhand, India; upadhyay91shashi@gmail.com; 3 Department of Pharmacognosy, Faculty of Pharmacy, Gazi University, 06330 Ankara, Turkey; 4 G. B. Pant National Institute of Himalayan Environment and Sustainable Development, Garhwal Regional Centre, Srinagar-246174, Uttarakhand, India; 5 School of Health and Biomedical Sciences, Discipline of Laboratory Medicine, RMIT University, P.O. Box 71, Bundoora, VIC 3083, Australiadaniel.dias@rmit.edu.au (D.A.D.); 6 Department of Pharmaceutical Technology, Avicenna Tajik State Medical University, Rudaki 139, 734003 Dushanbe, Tajikistan; shfarukh@mail.ru; 7 Phytochemistry Research Center, Shahid Beheshti University of Medical Sciences, Tehran 1991953381, Iran; taaheri.yasaman@gmail.com (Y.T.); navid.bp1994@gmail.com (N.B.); 8 Faculty of Medicine, University of Porto, Alameda Prof. Hernâni Monteiro, 4200-319 Porto, Portugal; ncmartins@med.up.pt; 9 Institute for Research and Innovation in Health (i3S), University of Porto, 4200-135 Porto, Portugal; 10 Department of Clinical Oncology, Queen Elizabeth Hospital, 30 Gascoigne Road, Hong Kong, China; 11 Zabol Medicinal Plants Research Center, Zabol University of Medical Sciences, Zabol 61615-585, Iran

**Keywords:** α-pinene, β-pinene, pharmacological activities, cytotoxicity, bioavailability, clinical studies

## Abstract

α- and β-pinene are well-known representatives of the monoterpenes group, and are found in many plants’ essential oils. A wide range of pharmacological activities have been reported, including antibiotic resistance modulation, anticoagulant, antitumor, antimicrobial, antimalarial, antioxidant, anti-inflammatory, anti-*Leishmania*, and analgesic effects. This article aims to summarize the most prominent effects of α- and β-pinene, namely their cytogenetic, gastroprotective, anxiolytic, cytoprotective, anticonvulsant, and neuroprotective effects, as well as their effects against H_2_O_2_-stimulated oxidative stress, pancreatitis, stress-stimulated hyperthermia, and pulpal pain. Finally, we will also discuss the bioavailability, administration, as well as their biological activity and clinical applications.

## 1. Introduction

**Figure 1:**
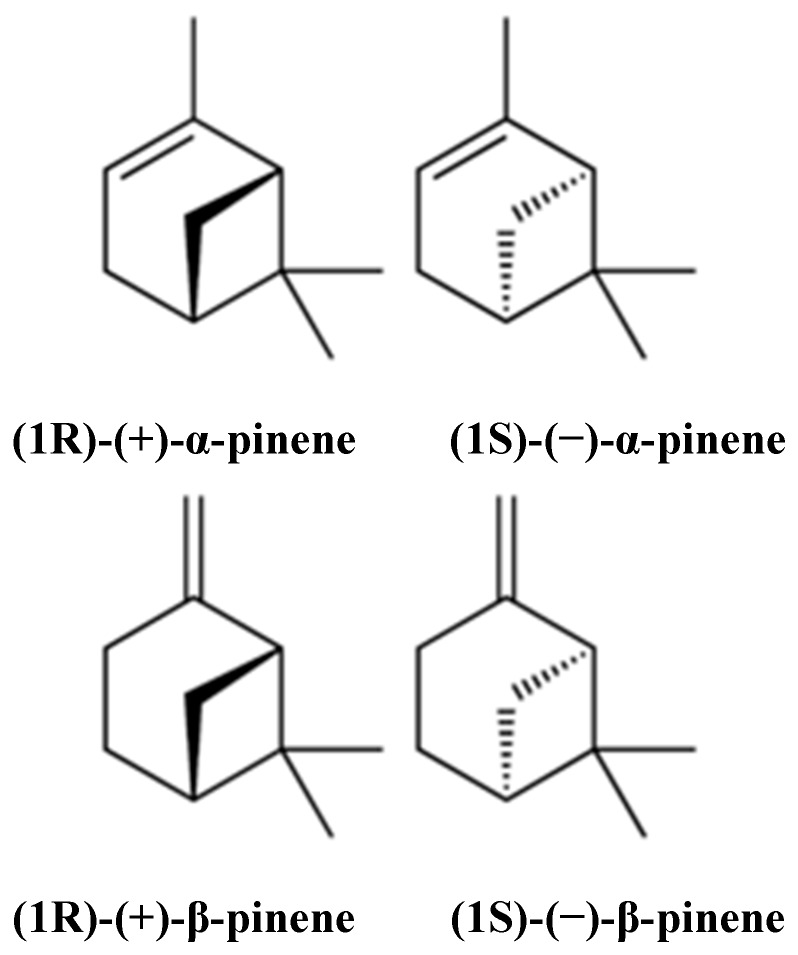
Chemical structures of α- and β-pinene.

Pinene (C_10_H_16_) is a bicyclic, double bond, terpenoid hydrocarbon [1]. α- and β-pinene are two isomers (Figure 1) found in nature, e.g., in pine (coniferous trees) essential oils (EOs). They are among the best-known representatives of a broad family of monoterpenes. α- and β-pinene enantiomers are different in their interactions with polarized light, and their mirror image does not overlap. Both structural isomers have two enantiomers (+) and (−). This difference yields four active isomers [2]. α-pinene is a colorless, water-insoluble but oil- and ethanol-soluble organic liquid. Its boiling point is 155 °C. α-pinene has been detected in at least 40 different EOs [2,3]. β-pinene is also a colorless organic liquid which is oil-soluble but ethanol- and water-insoluble. It has a boiling point ranging from 163–166 °C. It is obtained commercially by distillation or by α-pinene conversion [3]. It is also considered an essential intermediate in chilled dairy products, menthol, ionones, linalool, geraniol, citronellal, citral, citronellol, and candy production, but is mainly used in bakery products [2]. α- and β-pinene can be produced through biotransformation; some microorganisms, such as the fungi *Aspergillus* spp. and the bacteria *Pseudomonas* spp. have shown promising results in this regard. Pinenes have bicyclo [3.1.1] heptene or -heptane C-skeletons; thus, they take part in rearrangement and ring-opening reactions, producing different derivatives [4].

These two phytochemicals exhibit diverse biological activities, leading them to various applications and uses, such as fungicidal agents, flavors, fragrances, and antiviral and antimicrobial agents [5]. In addition, α- and β-pinene are components of renal and hepatic drugs [6]. Also, α- and β-pinene are used as antibacterials due to their toxic effects on membranes [7]. Moreover, α- and β-pinene have been found to have inhibitory effects on breast cancer and leukemia [8]. The application of pinenes goes beyond natural medicine; for instance, they have been proven to be very flexible in the synthesis of polymers [9,10,11,12]; polymers synthesized from pinenes are of better quality than other polymers [13]. The safety profile of pinenes is considered outstanding, allowing their use in various chemicals, and they are generally recognized as safe (GRAS) [14]. Thus, due to their physicochemical characteristics, it is challenging in the process of biotransformation, but are still used in the production of aroma compounds [15].

Some of the plants that contain or produce α-pinene, β-pinene, or both are: *Ocimum menthaefolium*, *Pinus* spp., *Juniperus communis*, *Rosmarinus officinalis*, *Lavandula stoechas*, *Coriandrum sativum*, *Cuminum cyminum*, *Juniperus oxycedrus*, *Myristica fragrans*, *Cinnamomum verum*, *Melaleuca alternifolia*, *Achillea millefolium*, *Ligusticum levisticum*, *Pistacia lentiscus*, *Grindelia camporum*, *Piper nigrum*, *Pilocarpus microphyllus*, *Agastache rugosa*, *Artemisia capillaris*, *Eugenia aromatic*, *Piper guineense*, *Solanum erianthum*, *Citrus limon*, *Citrus bergamia*, *Ferula kuhistanica*, and *Ferula clematidifolia* [16,17,18].

## 2. Preclinical Pharmacological Activities of α- and β-Pinene

Terpenoids, namely α- and β-pinene, are important bioactive compounds derived from the EOs of various plant species. However, few studies have assessed the preclinical pharmacological effects of both compounds (Table 1, Table 2, Table 3, Table 4, Table 5, Table 6 and Table 7).

### 2.1. Antibiotic Resistance Modulation

Bacterial pathogens have a great ability to acquire resistance against antibiotics; a serious problem or threat for both the medical and scientific communities. A report revealed that approximately 25,000 patients die annually in Europe due to multidrug-resistant bacteria infections [19].

Gastroenteritis is a disease caused by a multidrug-resistant bacterium known as *Campylobacter jejuni*. A report in the U.S. declared it as a serious threat to public health [20]. α-pinene has been used as an antibiotic resistance modulator for *C. jejuni* [21], acting on antibacterial resistance modulation and the prevention of antimicrobial efflux (detected by the insertion mutagenesis method); this characteristic was assessed using broth microdilution and ethidium bromide accumulation assays. DNA microarrays were also used to assess the *C. jejuni* adaptation to α-pinene, showing that it was able to modulate the antibiotic resistance in *C. jejuni* considerably by reducing the MIC value of ciprofloxacin, erythromycin, and triclosan by up to 512 times. Ethidium bromide was deposited in wild-type strain at higher degrees compared to antimicrobial efflux mutant, suggesting that α-pinene targets antimicrobial efflux systems.

On the other hand, Griffiths et al. recorded the effect of α-pinene on the growth of some microbes, i.e., *Nocardia* sp. strain (P18.3), *Pseudomona sputida* PX1 (NCIB 10684), *Pseudomonas* sp. strain PIN18 (NCIB 10687), and *P. fluorescens* NCIB 11671. Strains were cultured into agar slants containing α-pinene (3 g/L in media), and their growth was analyzed. *Nocardia* sp. (P18.3) growth in the basal salt culture medium with α-pinene was not notable, while fast linear growth was recorded in Erlenmeyer flask cultures from vapor tubes [22]. Besides this, *Pseudomonas* strains (NCIB 10684, 10687, and 11,671 and PL) grew quickly when α-pinene (0.3%, *v*/*v*) was added to the growth medium.

### 2.2. Anticoagulative Activities

*Angelica sinensis* is one of the most important herbs applied in traditional Chinese medicine as crude drugs for hematopoietic and anti-inflammatory activity for healing menstrual-related diseases [23]. Yang et al. studied *A. sinensis* and its constituents as an anticoagulative agent. Leaves were used for administration, and New Zealand White rabbits were used as model organisms in the study. Two α-pinene derivatives were extracted from the overground parts (10 g) of *A. sinensis.* Thrombin time (TT) and Platelet aggregation (turbidimetric method) assays were used to assess the in vitro anticoagulative properties. Results demonstrated that α-pinene derivatives prolonged thrombin time somewhat and severely prevented platelet aggregation. These α-pinene derivatives were found to be potent anticoagulative agents, and it was stated that these compounds were able to prevent thromboxane A_2_ production or platelet Ca^2+^ promotion [24].

### 2.3. Antitumor Activity

Tumor is a disorder in the growth and development of cells normally categorized by excess or abnormal cells multiplication. There are two types of tumors, commonly known as benign and malignant tumors. Among all types of malignant tumor cells (cancerous cells), lung tumor is generally the most diagnosed form of tumor worldwide, causing mortality of almost 1.38 million people each year [25,26]. It is roughly classified into two types: small cell lung carcinoma (SCLC) and non-small cell lung carcinoma (NSCLC). NSCLC is the most common and aggressive but with few limited treatment options. Survival rate of the NSCLC cases is low. Paclitaxel and carboplatin are commonly used anticancer drugs, with various side effects, such as leucopenia, hepatic and renal function disorders, nausea, and vomiting [27]. Such kinds of side effects have been minimized by coupling the drug with non-toxic enhancing agents (terpenoids with low molecular weight found in EO) and anticancer drugs [27,28,29]. α-pinene is an important terpenoid with anticancer properties against human ovarian cancer cell lines, hepatocellular liver carcinoma cell lines, and N2a neuroblastoma cells [30,31,32]. Synergistic anticancer effects have been demonstrated by α- and β-pinene against NSCLC with paclitaxel (an anticancer drug) using the combination index method and isobologram investigation [27]. A study revealed that α- and β-pinene do not exhibit considerable effects distinctly; however, when coupled with PAC, both constituents increase PAC-stimulated mitotic cell cycle arrest and apoptosis [27].

α-pinene has been shown to stimulate apoptosis proved by initial disruption of mitochondrial function, ROS formation, improved caspase-3 properties, heterochromatin aggregation, DNA disintegration, and exposure of phosphatidyl serine on the cell surface [33]. Additionally, the environment is supposed to significantly minimize cancer growth, and this was evidenced by Kusuhara et al., who stated that rats placed in fragrant environment with α-pinene exhibit a decrease in melanoma growth [34]. However, direct α-pinene did not display any *in vitro* effect on melanoma cell production. Chen et al. evaluated the inhibitory action of α-pinene on hepatocellular carcinoma BEL-7402 cells in vitro and in vivo using MTT assay [35,36]. α-pinene prevented BEL-7402 cells through cell cycle arrest at G2/M phase, down-regulated Cdc25C mRNA and protein expression, and decreased the action of cycle dependence on kinase 1 (CDK 1). Other studies have also suggested the use of α-pinene as a potent antitumor drug. Yang et al. measured the inhibitory properties of α-pinene on human hepatocellular carcinoma (HepG2) cells propagation, and found cell cycle arrest at G_2_/M phase, which seemed to be associated with down-regulation of miR-221 expression and up-regulation of CDKN1B/P27 and CDKN1C/P57 expression [37]. The effect of α-pinene on cell cycle regulation in HepG2 cells was also investigated and developed as a promising chemotherapeutic drug for administration in hepatocellular carcinoma [38]. Zhao et al. tested the inhibitory potential of α-pinene on human prostate cancer in a mouse xenograft model. β-pinene-based thiazole derivatives were used as anticancer agents via mitochondrial-facilitated apoptosis. Three human cancer cell lines, namely, cervical carcinoma HeLa cells, colon cancer CT-26, and hepatocarcinoma SMMC-7721 cells were used in the study [39]. Out of all derivatives, the compound 5g was found to inhibit cell proliferation by stimulating cell cycle arrest in HeLa cells by ROS-mediated mitochondrial dysfunction signaling pathways [40].

### 2.4. Genomic Instability

Catanzaro et al. studied the action of α-pinene on genomic instability in Chinese hamster cell line (V79-Cl3). Cells were cultured in Dulbecco’s Modified Eagle Medium (DMEM) with fetal calf serum, penicillin, and streptomycin. Different doses of α-pinene (0, 25, 30, 35, 40, and 50 µM) were used to expose cells (3 × 10^5^ per dish) for 1 h. Cytotoxicity was assessed using the standard approach. Morphological study demonstrated considerable growth in micro and multinucleated cell frequencies. Apoptotic cells were seen at 40 and 50 µM. α-pinene stimulated genetic instability, inhibiting mitotic process and leading to irregularity in 50% cells. It was established by flow cytometry that α-pinene stimulated oxidative stress and DNA destruction [41].

### 2.5. Cytogenetic and Oxidative Effects

Coniferous plants-derived EO are mostly composed of α-pinene. Türkez and Aydin (2013) investigated the cytogenetic and oxidative activities of α-pinene on human blood cells. Cultured human blood cells were supplemented with varying doses (0, 10, 25, 50, 75, 100, 150, and 200 mg/L) of α-pinene for 1–2 days. Lactate dehydrogenase (LDH) and MTT assays demonstrated that α-pinene at 200 mg/L reduced cell viability. Further, no changes were recorded significantly in the rates of genotoxicity endpoints. However, total antioxidant capacity (TAC) and total oxidative stress (TOS) levels revealed dose-dependent changes. TAC levels enhanced post-administration of 25 and 50 mg/L of α-pinene, while TOS levels were reduced only at 200 mg/L α-pinene on human lymphocytes [42].

### 2.6. Gastroprotective Effect

Gastrointestinal transit is the time it takes food to leave the stomach and travel through the intestines. It is a crucial procedure affected by many parameters that takes a long time. For healing, various medicinal plants and their compounds, such as monoterpenes (α- and β-pinene), have been used as an important source of therapeutic agents for gastrointestinal disorders [43,44]. In a study, *Eucalyptus tereticornis*-derived EO and its components, like α- and β-pinene, were used as gastroprotective agents [45]. Gastric emptying was measured after supplementation of a liquid test meal containing phenol red at varying time intervals. Gut was divided into consecutive segments. Small intestine transit was analyzed, and liquid test meal was supplemented. Dye retention was determined using spectrophotometric methods. Isometric contractions were observed by isometric transducers and data acquisition system. *E. tereticornis* EO and its components reduced gastric retention in mice, and α- and β-pinene enhanced gastric tonus in anesthetized mice. α- and β-pinene have been found to contract gastric strips in vitro, to relax the duodenum. In contrast, *E. tereticornis* soothes the gastric and duodenal strips. *E. tereticornis* speeds up the gastric emptying of liquid, and part of its effect is related to the contrast action stimulated by α- and β-pinene on gut.

The gastroprotective and antiulcerogenic properties of α-pinene were also studied using Swiss mice. Different doses (10, 30, and 100 mg/kg) of absolute ethanol and indomethacin were used to stimulate the gastric ulcer. Gastric lesions were analyzed by determining the area of lesions using the Scion Image programme. Stomach samples were crushed and sandwiched for further testing. Acute gastric lesions were introduced into the Swiss mice, and these mice fasted for 12 h. Rats were administered with 0.5 mL of vehicle (0.1% tween-80 aq. solution), ranitidine (40 mg/kg), and α-pinene (at 10–100 mg/kg), dissolved in vehicles separately. Rats were sacrificed by cervical dislocation followed by stomachs removal and scanning, with the area of lesions being determined. Gastrointestinal transit ratio was also analyzed. Findings from the study displayed that pretreatment with α-pinene cased a decline in ethanol-induced gastric mucosa lesions. α-pinene provides similar gastroprotective effects for absolute ethanol-stimulated ulcers to ranitidine (40 mg/kg) [46]. Oral pretreatment with α-pinene did not exhibit any considerable influence on indomethacin-sensitized ulcer lesions. Moreover, no considerable variation was recorded between the lesions area of α-pinene and vehicle pretreated rats. However, ranitidine decreases the area of lesions more than alternatives.

Likewise, the antiulcer properties of oleoresin (*Pistacia atlantica*) and α-pinene were studied on Wistar strain male albino mice [47]. Compounds were isolated from oleoresin using GC-MS. Various doses (250–2000 mg/kg b.w.) of EO were given to eight selected mice. Toxicity (restlessness, dullness, and agitation) was recorded in mice after 72 h of administration. Then, 80% ethanol was supplemented. Mice were sacrificed after 2 h, and stomachs were detached. Gastric ulcers were determined using microscope. The antibacterial properties of cultured *Helicobacter pylori* strains were screened using disc diffusion assay. Up to 2000 mg/kg EO was found safe. EO exhibited activity against *H. pylori* strains, with the inhibition zone being found maximum for the clinical strain No. 5. The EO from selected plants exhibited MIC values between 275 and 1100 μg/mL. Ethanol was used to stimulate gastric ulcers, and screening was performed against varying doses of EO and Tween 80 in water (control). Results indicated that varying doses of EO (25, 50, and 100 mg/kg) considerably reduced ethanol-induced peptic ulcers. Histopathological analysis revealed that EO reduced ethanol-induced gastric tissue damage and necrosis.

### 2.7. Anxiolytic-Like Effects

Yang et al. evaluated the non-rapid eye movement sleep/anxiolytic/hypnotic behavior in a rat model. Electroencephalogram (EEG) and electromyogram (EMG) analysis were used to investigate both behavior and hypnotic effect during sleep. α-pinene and zolpidem were orally pre-supplemented, followed by pentobarbital (45 mg/kg) injection. Ex vivo electrophysiological measurements from brain slices and *in silico* molecular modeling or molecular docking were performed. α-pinene displayed sleep-increasing behavior by direct binding to GABA_A_-Benzodiazepine receptors (GABA_A_-BZD) and forcing as a partial modulator at BZD binding site. Varying doses (12.5, 25, 50, and 100 mg/kg, orally) of α-pinene considerably reduced sleep latency and enhanced NREMS duration without any consequences on REMS and delta action. Results from the study indicated that the hypnotic action of α-pinene on rats could be due to its modulation of GABA_A_-BZD receptors, similar to zolpidem [48].

Similarly, the effect of α-pinene on emotional behavior, deposition, and expression of related mRNA was investigated in rat brain [49]. Rats were exposed to α-pinene (10 μL/L air), and water was used as negative control for 60/90 min. After inhalation, quantitative measurement of α-pinene in brain and gene expression by RT-PCR was performed. EPM analysis was conducted to determine the anxiolytic-like action on rats after α-pinene inhalation for 60 or 90 min. Distance was considerably enhanced when rats inhaled α-pinene for 60 min longer than the control (water). No notably variation was recorded in the total distance traveled for 90 min. α-pinene inhalation for 60 min in brain was considerably enhanced compared to 90 min. BDNF mRNA expression in olfactory bulb and hippocampus was similar at 60- and 90-min inhalation. TH mRNA expression in middle brain at 60 min inhalation was remarkably enhanced in comparison with the control.

### 2.8. Neuroprotective Activities

Several neurodegenerative diseases, like Alzheimer’s and Parkinson’s, are triggered by oxidative imbalance [50]. EO of various plant species are able to reduce ROS formation. Excessive ROS production led to injuries in brain functioning; thus, defense against ROS-stimulated injury is vital for appropriate brain functioning, and it diminishes the likelihood of the development of neurodegenerative disorders. With this background, Porres-Martínez et al. investigated the action of α-pinene on H_2_O_2_-sensitized oxidative stress, using mice pheochromocytoma cells (PC12) as model. Antioxidant and protective effects of α-pinene were assessed for H_2_O_2_-sensitized oxidative stress in PC12 cells. Cell viability was loosed, and cell morphology was altered after pre-treatment with α-pinene. Intracellular ROS production was prevented by α-pinene; however, it increased catalase, superoxide dismutase, glutathione peroxidase, glutathione reductase, and hemeoxygenase 1, expression. α-pinene also played an important role in reducing apoptosis; thus, it was concluded that these monoterpenes have ability to defend the nervous system [50].

### 2.9. Cytoprotective Activity against H_2_O_2_-Stimulated Oxidative Stress

Excessive ROS (e.g., hydroxyl radical, superoxide anion, and hydrogen peroxide) formation unbalances cellular redox, leading to oxidative stress [51] and randomly oxidizing biological molecules (i.e., peroxidation, mitochondrial dysfunction, protein carbonylation, and DNA strand breaks and base damage) [52]. ROS overproduction protection and cellular redox equilibrium may be promoted by using several natural antioxidants and EO. *Salvia lavandulifolia* is a species used in traditional medicine to improve memory and cure several diseases. Porres-Martínez et al. studied the constituents of *S. lavandulifolia* in human astrocytoma 373-MG cell line as a cytoprotective agent against H_2_O_2_-stimulated oxidative stress. Doses ranged from 10 to 250 mM α-pinene. H_2_O_2_ significantly reduces cell viability by over 60%. However, pre-supplementation with α-pinene (10, 25, 50, and 100 mM) considerably enhanced cell viability in a dose-dependent fashion (IC_50_ = 79.70 mM). Pre-administration with α-pinene protected U373-MG cells from H_2_O_2_-stimulated oxidative damage, through blocking the loss of cell viability (IC_50_: 79.70 mM to α-pinene) and cell morphology, preventing ROS formation and lipid peroxidation and enhancing the endogenous antioxidant status by enhancing glutathione, CAT, SOD, GR, and GPx activities, and HO-1 properties and protein expression [51].

### 2.10. Inhibitory Effect on the Growth of Endocarditis Disease

Endocarditis is a disease triggered by cardiac wall or endocardium infection, promoted by different microbes, like bacteria, fungi, and chlamydia. Microorganisms belonging to *Streptococcus* and *Staphylococcus* genus, *Haemophillus parainfluenzae*, *H. aphrophilus*, *H. paraphrophilus*, *H. influenzae*, *Actinobacillus actinomycetemcomitans*, *Cardiobaccterium hominis*, *Eikenella corrodens*, *Kingela kingae*, and *K. denitrificans* are the major microbial agents causing endocarditis. In modern times, microbes have shown susceptibility to drugs used in various clinical therapies. Therefore, one study was attempted to slow down the growth of endocarditis, using monoterpenes like α- and β-pinene [53]. The MIC value was determined by solid medium diffusion method, whereas viable cells count was used to investigate the interference of MIC values on bacterial cell viability. *Staphylococcus aureus*, *S. epidermidis*, *S. pneumoniae,* and *S. pyogenes* strains were utilized for the proposed screening.

Phytochemicals, like α- and β-pinene, showed inhibitory activity against all bacterial strains studied. Some phytochemicals exhibited MIC values between 5 (α-pinene x *S. epidermidis* SSI 1, *S. pyogenes* and *S. pneumoniae*) and 40 μL/mL (β-pinene x *S. epidermidis*). In addition, some bacterial strains showed resistance to antibiotics, mainly gentamicin. *S. aureus* exhibited resistance to α- and β-pinene.

### 2.11. Antimicrobial and Antimalarial Effects

The antimicrobial activity of α- and β-pinene against bacterial (*E. coli*, *S. aureus*, and *Bacillus cereus*) and yeast (*Candida albicans*) strains was already assessed using disc diffusion method [54], and the results revealed that (+)-β-pinene possess approximately 2 to 12 times higher activity than (+)-α-pinene against both gram-positive and -negative bacteria, as well as *C. albicans*. The peel and major constituents, like α-pinene of 14 *Citrus* species EO, were evaluated against two bacteria, *Propionibacterium acnes* and *Staphylococcus epidermidis* [55]. Limonene, myrcene, α-pinene, linalool, β-pinene, and α-terpinolene were the principal constituents isolated from citrus peel. Findings revealed that EO had prominent antibacterial properties against *P. acnes*. However, citrus EO showed activity against *S. epidermidis*. It is concluded that the EO antibacterial activity may be due to the occurrence of α- and β-pinene, which damaged the cellular integrity of these microorganisms. Silva et al. studied the antimicrobial properties of α- and β-pinene enantiomers against *C. albicans*, *Cryptococcus neoformans*, *Rhizopus oryzae*, and methicillin-resistant *S. aureus* (MRSA). The authors found that α- and β-pinene enantiomers exhibited MIC values ranging from 117 to 6250 µg/mL. Negative enantiomers did not exhibit any activity against microbes up to 20 µg/mL. α- and β-pinene enantiomers demonstrated higher activity against *C. albicans* than MRSA. Positive enantiomers revealed a good ability to kill 100% of *C. albicans* in 60 min; however, for MRSA, a 6 h period was required for total killing [5]. Dhar et al. also assessed the preventing action of α-pinene derivatives [(+) and (−)] against gram-positive (*Micrococcus luteus* and *S. aureus*) bacteria, gram-negative (*E. coli*) bacteria, and a fungus (*C. albicans*). Regarding the main findings, (+)-α-pinene exhibited modest action against the selected microbes, while (−)-α-pinene did not exhibit any properties. Among all studied α-pinene derivatives, maximum antimicrobial properties were demonstrated by β-lactams. Though (−)-α-pinene does not display antimicrobial properties, the derivatives (i.e., β-lactam, amino ester, and amino alcohol) exhibited antimicrobial properties [56]. The effect of β-pinene derivatives (25, 3-cyanopyridine) was also screened against four bacteria, namely, two gram-negative (*Klebsiella pneumoniae*, *Enterobacter aerogenes*), two gram-positive (*S. aureus*, *S. epidermidis*), and a fungus (*C. albicans*) [57]. MIC values of all studied derivatives screened against the studied microbes were found to be 15.6-125 mg/L. On the other hand, the EO and main constituent α-pinene obtained from *Bursera morelensis* stems were tested against *C. albicans* strains (ATCC 14065, ATCC 32354, donated strain, and CDBB-L-1003) [58]. Maximum anti-*Candida* effects were found to the EO and its constituent α-pinene. The minimal fungicidal dose of EO was 2 mg/mL. Based on survival kinetics, it was found that *C. albicans* population was slightly reduced after 12 h.

Macêdo et al. analyzed the (+)-β-pinene enantiomers and their antifungal properties against *Candida* spp. strains. Results demonstrated that MIC values ranged from <56.25 to 1800 µmol/L to (+)-β-pinene. After adding ergosterol, there were no changes in the MIC value of (+)-β-pinene; however, it changed with the addition of sorbitol. Molecular docking simulations revealed higher interaction with delta-14-sterol reductase (−51 kcal/mol). (+)-β-Pinene showed anti-biofilm properties against several *Candida* spp. [59]. The positive enantiomers of α-pinene were also investigated for antibacterial effects against *S. aureus* and *E. coli* [60]. Inhibition zone of 11 mm was recorded for gram-positive bacteria at 160 µL/mL, while an inhibition zone of 12 mm was stated against the gram-negative strain. (+)-α-pinene (at 1.25 and 2.5 µL/mL) was able to remove bacterial colonies development at one time of exposure of *E. coli* strain for 2 h.

The antimalarial activity of α- and β-pinene was already assessed, being stated that (+)-α-pinene has 250 times higher antimalarial properties than (+)-β-pinene. When looking at antioxidant activity, the authors stated that terpenes have greater antioxidant activity than other compounds, like ascorbic acid [54].

### 2.12. Anti-Leishmania Activity

Rodrigues et al. studied the anti-*Leishmania* effects of *Syzygium cumini* leaves EO and its principal constituents, α-pinene, in Swiss rats. The anti-*Leishmania* effects were assessed using MTT methods, and macrophages cytotoxicity was also measured. Results demonstrated that α-pinene exerts cytotoxic effects at varying degrees of death percentages (93.7%, 83.2%, and 58.4%), directly correlated with the different doses (100, 50, and 25 mg/ml, respectively) used against promastigotes of *Leishmania amazonensis* [61].

### 2.13. Effect on Cytochrome P-450 Levels

The metabolism of endogenous compounds, like hormones, fatty acids, and prostaglandins, and so on, involves the mono-oxygenase system mediated by cytochrome P-450 [62]. Cytochrome P-450 is an important protein family, with a key role in detoxification or activation of several hydrophobic foreign compounds, drugs, carcinogens, and environmental pollutants [63]. Austin et al. assessed the effect of pinene on cytochrome P-450 level in liver of Sprague–Dawley mice. Mice were placed in cages at a constant room temperature, with 12 h dark and 12 h light period, with food and water. Diverse terpenoids, namely camphor, limonene, menthol, myrcene, or pinene, dissolved in 10% ethanol AND 90% corn oil (at 40 mg/kg b.w.), were injected three times into healthy mice. Comparative assessment of exposed mice was performed with other mice administered with phenobarbital (0.9% NaCl) and control mice supplemented with vehicle (10% ethanol and 90% corn oil). Mice were sacrificed by cervical dislocation, 18 h after administration of the third dose. Liver microsomal vesicles were finally separated, and cytochrome P-450 was studied using CO-reduced difference spectroscopy. No visible changes were recorded on membrane proteins of liver microsomal of mice followed by administration of different terpenoids. Radioimmunoassay was used to calculate cytochrome P-450 levels in microsomal membrane vesicles after administration. The P-450 amount in control mice was 0.05 mM/mg, and in phenobarbital-administered mice was 1.02 mM/mg, demonstrating a 23-fold stimulation of PB P-450. These data demonstrate that different terpenoids possess considerable stimulation of PB P-450 in mice [62].

### 2.14. Effect on Pancreatitis

Bae et al. evaluated the effect of α-pinene on acute pancreatitis (AP) in C57BL/6 female mice. Mice were bred and placed in standard cages in a room temperature-controlled room and were kept in the dark and light (12 h each) with food and water. AP was induced in mice followed by fasting for 18 h after administration of cholecystokinin-like cerulein (50 µg/kg, i.p.) injection every hour (for 6 h). Varying doses (5, 25, or 50 mg/kg) of α-pinene were injected. Rats were sacrificed at the end of the final cerulein injection. Blood samples were investigated for measurement of serum and lipase activity. Morphological analysis and scoring were performed by rapidly removing pancreas of all rats. Neutrophil sequestration (by measuring tissue myeloperoxidase (MPO) as an indicator), reverse transcription PCR, and real-time PCR were performed. Cerulein-stimulated AP altered body weight and pancreatic weight of rats. Body weight (BW) was reduced, and pancreatic weight (PW) was attained due to edema, thus increasing the PW/BW proportion. But PW/BW proportion was reduced followed by α-pinene stimulation. Serum lipase and amylase levels were markedly increased during cerulein-stimulated AP, while α-pinene decreased lipase and amylase levels [64].

### 2.15. Porphyrogenic Properties

Acute porphyrins are one of the few in-borne errors or genetically determined diseases in humans. In some females, it is triggered during puberty or menstrual cycles and can be precipitated by a variety of nutritional and environmental features, such as drugs and xenobiotics. Terpenes also exhibit this kind of activity. Bonkovsky et al. screened the effects of camphor, thujone, and pinene as potent hepatic porphyrogens. The authors still focused only on the action of α-pinene on Barred Rock Chickens [65]. The livers of 16- to 18-day-old chicken embryos were selected, cultured, and compared with white Leghorn embryos to determine behavior. Porphyrins were fluorometrically measured. Spectrophotometric methods were used to determine ALA synthase and heme oxygenase enzymes. Cultured sonicated cells were used for Western blotting analysis. α-pinene formed few extensions of porphyrins in liver cells of chick embryo. α-pinene also led to deposition of 100–150 porphyrins/mg (copro- and protoporphyrins) proteins at the highest screened dose (1 mM). These findings suggest that the action of α-pinene on hepatic heme metabolism resembles those formed by glutethimide and other phenobarbital-like drugs.

### 2.16. Protective Effect Against Cytotoxicity

The cytoprotective effects of α-pinene were also screened. The α-pinene protective effect against cytotoxicity stimulated by aspirin was measured in IEC-6 cells of rats (small intestine epithelial cells). The antioxidant potential using DPPH free radical scavenging was also analyzed using varying doses of α-pinene (25, 50, 100, 200, 300, and 400 µg/mL), and ferric reducing activity (FRAP) was investigated. IEC-6 cells were matured in the DMEM. Cell morphology was examined using inverted microscope. SOD, mitochondrial SOD, and glutathione action were investigated using standard assays. Findings from the study revealed that the anti-DPPH properties increased with increasing α-pinene doses until a maximum dose (400 µg/mL). FRAP properties were enhanced with the raise in α-pinene dose (up to 300 µg/mL). Cultured cells were administered with aspirin (10 mM), followed by incubation with varying doses of α-pinene (0.5, 1, 1.5 µg/mL) for 24 h. No effects were observed on cell viability using the lower dose of α-pinene. A significant increase in IEC-6 cell viability was recorded on aspirin exposure with α-pinene, compared to aspirin exposure alone. Aspirin displayed a negative alteration on cell morphology, but aspirin exposure with α-pinene was unable to stimulate morphological changes [66].

Karthikeyan et al. determined the protective action of α-pinene on UVA-stimulated oxidative imbalance in human skin epidermal keratinocytes (HaCat cells). HaCat cells were preserved in DMEM supplement and divided into four groups, i.e., non-irradiated control cells, α-pinene (30 µm) administered cells, UVA (10 J/cm^2^) irradiated cells, and α-pinene pre-administered (30 min before) and UVA irradiated cells. Cellular injury was triggered UVA-irradiation (10 J/cm^2^) in the presence and absence of α-pinene. Cytotoxic effect was determined using MTT assay [67]. ROS production, change in mitochondrial membrane potential (MMP), and DNA damage were investigated using fluorescence microscopy and spectrofluorometry. Role of α-pinene against cytotoxic effect was measured using Western blot method. Results indicated that up to 30 µm of α-pinene, HaCat cell death was not verified. HaCat cell viability was considerably decreased after UVA exposure, but UVA-stimulated cytotoxicity was inhibited by α-pinene pre-administration. Findings suggested that UVA radiation enhanced ROS formation. However, α-pinene pre-administration remarkably blocked ROS formation. UVA-exposed cells exhibited enhanced levels of peroxidation, compared to control cells. UVA-stimulated lipid peroxidation level was inhibited by α-pinene. Likewise, the protective effect of α-pinene in Swiss Albino mice was measured on UVA-sensitized cytotoxicity (skin photoaging) [68]. Cellular injury was triggered by UVA-irradiation (10 J/cm^2^ per day) for 10 days. Before exposure, rats were administered with α-pinene (100 mg kg/b.w.). Antioxidant enzymes and oxidative imbalance was determined using biochemical methods. Histopathological analysis was also performed in mice skin. Apoptotic protein expression was investigated using western blotting assay. Antioxidant enzymes (SOD, CAT, GPx, and GSH) levels were considerably reduced in UVA-exposed rat skin. High levels of ROS formation were detected, injuring lipid-rich cell membranes. Peroxidation level was higher in UVA-exposed rat than in non-irradiated control and α-pinene alone-administered rats. α-pinene supplementation prior to UVA exposure remarkably enhanced the concentration of antioxidant enzymes compared to non-irradiated control, and considerably decreased lipid peroxidation.

### 2.17. Allergic Rhinitis

The α-pinene effects were investigated in allergic rhinitis in BALB/c female rats [69]. Varying doses (0.1, 1, and 10 mg/kg) of α-pinene were administered daily to rats for 10 days, 1 h before or 1 h after intranasal OVA challenge. HMC-1 cells were cultured into IMDM medium. HMC-1 cells were administered with α-pinene (0.1, 1, and 10 µg/mL) for 1 h. Western blot was performed to determine the protein expression. Histological analysis was also performed. Results showed that prior administration with α-pinene reduces clinical symptoms, i.e., reduced number of nasals, eye, and hearing fractures; spleen weight; and decline in interleukin-4 level and reduction in nasal immunoglobulin E level in OVA-sensitized rats.

### 2.18. Anticonvulsant Effects

Epilepsy is the oldest and third most common disorder among neurological disorders known in humans, after stroke and Alzheimer’s disease [70]. Despite that fact that several anti-epileptic drugs have been discovered, most patients have not responded to these, as epilepsy may be related to oxidative imbalance [71]. Various medicinal plants as well as drugs have been found to cure oxidative imbalance. Zamyad et al. used the leaves and flowers of *Ducrosia anethifolia* and their main constituent α-pinene as anticonvulsant agents in Wistar mice. Mice were administered with EO (500 mg/kg). Death and illness in mice were measured. Pentylenetetrazole (PTZ, 80 mg/kg) was injected to stimulate convulsions in mice. Diazepam (2 mg/kg), EO (25–200 mg/kg), and α-pinene (0.2 and 0.4 mg/kg) were supplemented 30 min before receiving PTZ. Mice behavior was observed with a CD camera [70]. Oxidant and antioxidant attributes were measured using the temporal lobes of dissected brain. Results demonstrated that EO exhibited action against PTZ-stimulated seizures, being able to markedly decrease mice convulsion. Death rate and PTZ-stimulated seizures were significantly reduced following EO (50–200 mg/kg) and α-pinene (0.2 and 0.4 mg/kg) administration. Both EO and α-pinene were capable to reduce the oxidative imbalance. Likewise, Felipe et al. studied the anticonvulsant properties of α- and β-pinene on male Swiss Albino rats (*Mus musculus*). Initially, α- and β-pinene (2000 mg/kg) were administered to rats and examined for 10–240 min, and for 14 consecutive days. Rats were administered with vehicle (0.5% Tween 80 in saline), α-pinene (100–400 mg/kg), β-pinene (100–400 mg/kg), equimolar mixture of α- and β-pinene (400 mg/kg), and diazepam (2 mg/kg). After 1 h supplementation, PTZ (80 mg/kg, i.p.) was injected in pretreated rats to stimulate seizures. Then, rats were sacrificed by cervical dislocation, and brains hippocampus and striatum were removed immediately for neurochemical measurement. LD_50_ determined for α- and β-pinene was higher than 2000 mg/kg. Although mild sedative effects were recorded, no mortality was stated at this dose. Rats were administered with different compounds and subjected to PTZ-stimulated convulsion. At 400 mg/kg, compounds considerably reduced seizure intensity. Latency of the first convulsion was remarkably enhanced with the mixture (400 mg/kg) of α- and β-pinene. β-pinene and mixture (400 mg/kg) considerably enhanced the time of mortality of rats. α-pinene and equimolar mixture significantly decreases the level of hippocampal nitrite and striatal content of dopamine and norepinephrine [72].

### 2.19. Anti-Inflammatory and Analgesic Properties

Inflammation is an immune reaction that provides defense to the human body against damage or infection [73]. Inappropriate immune responses in body often lead to inflammatory diseases. Macrophage plays an essential role in inflammatory reactions. Rufino et al. analyzed the anti-inflammatory and chondroprotective effect of *Juniperus oxycedrus* EO and their major constituents α-pinene enantiomers in human chondrocyte [74]. (+)-α-pinene (1) exhibited the strongest prevention against IL-1β-stimulated inflammatory and catabolic pathways. Similarly, in another study, the anti-inflammatory effect of α-pinene was studied on peritoneal macrophages of male C57BL/6 rats [73]. Results revealed that α-pinene do not exhibit cytotoxicity up to 20 µL/dose. α-pinene reduced IL-6 and TNF-α formation in macrophages of rats. It was also found that nitrite production was reduced by α-pinene. In addition, western blot analysis revealed that α-pinene prevented NF-kB promotion. Likewise, Li et al. analyzed the activity of α-pinene and Frankincense oil (*Boswellia carterii*) on inflammation in Kunning rats. Frankincense oil, water extracts, and their constituents were screened against xylene-stimulated edema and formalin-sensitized hind paw edema in mice model to assess the anti-inflammatory and anti-analgesic properties. Frankincense oil revealed higher anti-inflammatory and anti-analgesic effects, compared to mice administered with water extracts. Strong pharmacological effects were observed using the combination of the three separated constituents in hind paw inflammation and COX-2 overexpression, when compared with their use alone [75].

### 2.20. Effect on Stress Stimulated Hyperthermia

The effect of α-pinene on stress-stimulated hyperthermia in Wistar mice was analyzed [76]. Rats were exposed to varying doses (0.003%, 0.03%, and 0.3%) of α-pinene odor. At a constant room temperature, the mice were kept in cages at 12 h dark and 12 h light with food and water. Followed by exposure to odor of different doses of α-pinene, all mice were exposed to different unknown environments. Behavioral changes, like sniffing, rearing, grooming, resting, and common activities, were recorded. Body temperature change (abrupt enhancement) was recorded in 0.03% α-pinene after transferring mice from home cage. However, α-pinene odor at doses of 0.003% and 0.3% decreased stress-stimulated hyperthermia in mice. Exposure at doses of 0.003% and 0.03% did not change heart rate, whereas 0.3% led to alterations. Finally, the study indicated that only the dose of 0.03% exerts inhibitory effects on stress-stimulated hyperthermia. Further, the dose of 0.3% reduced cardiac responses; thus, it was concluded that varying doses of α-pinene bind to diverse olfactory receptors and stimulate different types of neuronal events.

### 2.21. Permeability Glycoprotein (PgP) Transporter

Green et al. studied the impact of α-pinene on permeability glycoprotein (PgP) in wood mice (*Neotoma* species). Selected wood mice were sacrificed and gut was removed quickly from the stomach. Further analysis in the laboratory was performed. Findings of this study revealed that α-pinene is not a PgP substrate [77].

### 2.22. Effect on Pulpal Pain (Dental Pain)

The impact of α-pinene on pulpal pain in Wistar mice was investigated [78]. Selected mice were cannulated by their lateral ventricles for capsaicin (100 µg) supplementation. Varying doses (0.1, 0.2, and 0.4 µM) of α-pinene were administered by injection. Anti-inflammatory effect of α-pinene was checked by evaluating the COX-2 expression in the sub nucleolus caudalis (Vc) of mice. α-Pinene (0.2 and 0.4 μM) was able to reduce nociception. The expression of COX-2-positive cells in the Vc of capsaicin-treated mice was considerably enhanced, which was prohibited by 0.4 μM α-pinene.

### 2.23. Sensory Irritation

The effects of α- and β-pinene enantiomers were analyzed on expiratory and inspiratory airflow (V) in OF1 (I.O.P.S. Caw) and KTL [(Hsd/Ola): NIH/(SPF)] male rats [79]. Rats were placed in steel cages. After that, rats were kept in a glass tubes and exposed (15 min) to the selected α- and β-pinene enantiomers. Inspiratory (VI) and expiratory (VE) airflows were investigated using a differential pressure transducer attached with pneumotachograph. RD_50_ (f) values were analyzed. Initially, rats were kept in the room air, but no irritation was recorded. However, followed by pinene enantiomers’ exposure, irritation was recorded, which suggested that D-enantiomers were the potential sensory irritants. RD_50_ for α- and β-pinene d-enantiomers were nearly similar. Nielsen et al. also measured the α-pinene enantiomers effect on acute inhalation in BALB/c rats. α-pinene was vaporized and diluted with room air. Sensory irritation was recorded on upper respiratory tract by (+) enantiomer during the exposures at 100–369 ppm. The threshold dose was 70 ppm, which is almost equal to the non-effective dose (40 ppm) in humans. 200 ppm and higher doses were noteworthy for airflow limitation. However, no irritating action was recorded at alveolar and CNS levels [80].

### 2.24. Toxicokinetic Effect

The influence of α-pinene uptake, distribution, and elimination in pulmonary function was assessed in human volunteers [81]. In an exposure chamber, human volunteers were exposed by inhalation (2 h, 50 W) to α-pinene (10–450 mg/m^3^). The total pulmonary uptake of α-pinene was measured by calculating the difference between the total amount of inhaled and exhaled solvent in air. Capillary blood, urine, and exhaled air were measured after exposure. Absolute α-pinene uptake increased linearly with exposure dose. The α-pinene dose initially increased arterial blood during exposure, and then stabilized at the end of exposure. Few undesirable consequences were recorded during the exposure, such as eyes, nose, and throat irritation. These responses were found to be statistically significant. Prior to exposure, lung functions of all subjects examined were normal. However, only minimum changes were recorded after exposure, such as larger bronchial diameter than bronchoconstriction, but these changes did not exhibit a strong relationship.

### 2.25. Treatment of IgA Nephropathy

Piperazine ferulate tablets combined with eucalyptol, limonene, and pinene enteric soft capsules were used for IgA nephropathic children therapy by Liu et al. [82]. In the observation group, patients were treated with piperazine ferulate tablets (0.1 g/dose and 3 times/day) combined with eucalyptol, limonene, and pinene enteric soft capsules (0.1 g/dose and 2 times/day) for 6 months. IgA was detected by ELISA, and fibronectin and complement C3 were detected by single radial immunodiffusion. The effective rate of observational group (12 patients) was significantly higher when compared with hormone group (18 patients). Differences between serum IgA, fibronectin, and complement C3 of two selected groups were not statistically significant [82].

### 2.26. Insecticidal or Larvicidal Effects

EO of *Plectranthus barbatus* leaves and α-pinene were used to determine their impact on larvae of malaria (*Anopheles subpictus*), dengue (*Aedes albopictus*), and Japanese encephalitis (*Culex tritaeniorhynchus*) mosquito vectors [83]. GC and GC-MS were performed to analyze the main EO constituents. Dog biscuits and yeast powder (3:1 proportion) were used to feed larvae. Larvicidal properties of EO (40, 80, 120, 160, and 200 µg/mL) and their components, like eugenol, α-pinene, and β-caryophyllene (12–100 µg/mL each), were measured using WHO protocol. Larvae death was observed at 24 h post-administration. EO displayed considerable larvicidal properties with LC_50_ values of 84.2, 87.2, and 94.3 µg/mL for the selected mosquito species. For *Anapheles subpictus*, the three main components (eugenol, α-pinene, and β-caryophyllene) demonstrated potent larvicidal properties (LC_50_ = 25.5, 32.1 and 41.7 μg/mL) followed by *Aedes albopictus* (LC_50_ = 28.1, 34.1, and 44.8 μg/mL, respectively) and *Culex tritaeniorhynchus* (LC_50_ = 30.8, 36.8, and 48.2 μg/mL, respectively).

## 3. Bioavailability of α-Pinene and β-Pinene

According to the Codes of Federal Regulations (FDR), Title 21; Part 314 A, bioavailability is defined as the “rate and extent to which the active ingredient or active moiety is absorbed from a drug product and becomes available at the site of drug action” [86].

Various studies have described that α-pinene and β-pinene display antimicrobial [87], anticancer [33,36], anti-inflammatory [64], and antiallergic [69] properties. Nevertheless, most of these studies lack information concerning bioavailability and pharmacokinetics of the active terpenes. However, to take advantage of the bioactivity of a particular natural product, in this case *α*-pinene and β-pinene, and to further use it as either a supplement or drug in future, it is necessary to study the absorption, distribution, and metabolism

Monoterpenes are essentially metabolized by cytochrome P_450_ monooxygenases, epoxide hydrolases, and dehydrogenases to mono- and dihydroxylated substances, as well as higher oxidized metabolites that are conjugated basically to glucuronic acids [88]. According to the authors, after absorbing α-pinene, the proposed in vivo human metabolites are *trans*-verbenol (tVER), *cis*-verbenol (cVER), myrtenol (MYR), myrtenic acid (MYRA), αPNM3, and αPN-M1 [89]. In contrast, β-pinene metabolites from brushtail possum (*Trichosurus vulpecula*) were found to be myrtenic acid, present in their urine [90].

### 3.1. Dermal Application

Kohlert, et al. [91] reported the preparation of eucalyptus or pine oils enriched with α- and β-pinene, camphor, 3-carene, and limonene, which have been applied in most works investigating the dermal absorption of EO-based substances, as these terpenes increase percutaneous absorption of drugs and other compounds due to their lipophilic characteristics. According to Cal and Sopala [92], the maximum concentration of terpenes in the stratum corneum (SC) and epidermis (ED) was obtained within 15 min of application. It was also demonstrated that the extent of absorption depends on the size of treated skin area, skin properties, concentration of administrated compound, and time of exposure [91,92].

Cal and Sopala [92] investigated the ex vivo skin absorption of Vicks VapoRub^®^ (α-pinene 4.8%, β-pinene 1.1%) using human cadaver skin of a 40- to 50-year-old Caucasian women. An infinite dose of 100 mg/cm^2^ was applied on a 0.65 cm^2^ skin area; placed in a flow-through diffusion chamber; and left for 15, 30, and 60 min, respectively. For α-pinene, the maximum concentration C_max_ 40 µg/cm^2^ in the SC was within 15 min of application of Vicks VapoRub. β-pinene reported a C_max_ 290 µg/cm^2^ 60 min after application. Although their physicochemical properties were similar, their penetration profiles into SC differed. Though the β-pinene content in the product was 4.5 times lower than that of *α*-pinene, the total accumulation of β-pinene in the SC was seven times greater due to its strong lipophilic nature [92].

β-Myrcene, limonene, α-pinene, β-pinene, linalool, geraniol, citronellol, and isomenthone are typical monoterpene constituents of rose oil [93]. In a study investigating the in vitro percutaneous permeation of rose oil using human tissues, Schmitt, Schaefer, Sporer and Reichling [93] stated that α- and β-pinene had different permeability behaviors when they were applied as a neat solution compared to as a component in rose oil (rose oil constituent). When α- and β-pinene were applied as a neat, single substance, P_app_-values (apparent permeability coefficients in cm/s) were reported to be high (α-pinene 6.49 × 10^−5^ cm/s and β-pinene 4.48 × 10^−5^ cm/s) compared to other monoterpenes in rose oil applied as a neat substance. However, when pinene was available as a component of rose oil, the permeability of α-pinene dropped while the permeability of β-pinene was enhanced. The P_app_-value of β-pinene was approximately four times higher (5.76 × 10^−5^ cm/s) than that of β-myrcene, limonene, and *α*-pinene (1.43 × 10^−5^ cm/s). It is apparent that the skin permeability was positively or negatively affected depending on the other constituents available in the mixture [93].

### 3.2. Inhalation 

α- and β-pinene are insoluble in water yet soluble in blood and adipose tissues. Terpenes indicate high respiratory uptake and accumulation in adipose tissues [94]. In a study by Filipsson [95], the relative net uptake of α- and β-pinene was reported to be 62% and 66%, respectively (see Table 8).

The mean concentration of α-pinene indicated linear kinetics with increasing exposure concentration in blood, as well as in urine [95]. Blood concentration mainly depends on exposure time, dose, or exposure concentration in the chamber, and the presence of other monoterpenes in the treatment chamber. Terpenes are soluble in oil; so, they are expected to accumulate in adipose tissue and thus exhibit greater t_l/2_ in blood. A higher fat: blood partition coefficient for α-pinene resulted in an increase in distribution rate of *α*-pinene from blood to fat and a higher metabolic rate for α-pinene [95]. During the early stages of exposure, arterial blood concentration increased rapidly and then levelled off at the end of exposure. After the end of exposure, the decaying of pinene in blood was rapid and the author distinguished three log linear phases of the pinene concentration in arterial blood, namely, initial, middle, and last. The mean half time (t_1/2_) of the three phases identified—initial, middle, and last—were 4.8, 38, and 695–555 min, respectively. Likewise, the respective β-pinene t_1/2_s were 5.3 min, 41 min, and 25 h. The long half-time of α-pinene implies that it would take more than 2 days for the body to be almost totally cleared of α-pinene (estimated as 5 × t_1/2_) [95]. Therefore, β-pinene may take more than 2 days to completely clear the body. Blood clearance values measured up to 21 h were cl_21h_ 1.5 lkg^−1^h^−1^ for *α*-pinene and 0.5 lkg^−1^h^−1^ for β-pinene. Less than 0.001% of the total *α*-pinene dose was excreted unchanged in the urine within 0.5 h after exposure, according to Filipsson.

### 3.3. Oral Administration 

Schmidt and Göen [89] recruited 4 volunteers (receiving orally α-pinene, 9 mg) and studied the metabolic products, including *α*-pinene, namely, myrtenol (MYR), and *cis*- and *trans*-verbenol (cVER; tVER) levels in blood and urine. The maximum blood concentration c_max_ for MYR was 11 µM. tVER-26µM and cVER-9.3 were recorded and metabolites concentration reached their maxima (t_max_) 1.6 h after exposure. The maximum concentration of the urinary α-pinene metabolites was reached 1.6 h after exposure and then within 24 h; after it declined to pre-exposure levels with elimination half-lives of 1.5 h (MYR) and 1.6 h (cVER and tVER). The total eliminated amounts were 1.5% (MYR), 5.6% (cVER), and 4.1% (tVER) of the orally applied dose. The metabolic product, myrtenic acid (MYRA), showed renal elimination of 6.7% share of 9 mg oral dose at t_max_ 1.6 h after ingestion, and an elimination half-time of 1.4% [89]. Thus, the study showed that the blood concentration of orally administrated, unmetabolized *α*-pinene lasts for a short duration at low concentrations (<4 µg L^−1^) in blood and could be rapidly eliminated from the body unchanged via lungs. Only 22% (MYRA–7%, cVER–6%, tVER–4%, MYR–2%, αPN-M3–2%, and αPN-M1–1%) of the oral dose administrated was detected as metabolites in urine. The amount of α-pinene metabolites found in urine was notably greater compared to blood, with this being indicative of a fast transfer of metabolites from blood to urine with a rapid renal elimination. Detectable metabolite levels and *non*-detectable α-pinene in blood after low oral doses indicate a fast and approximately entire pre-systemic metabolism, such as hepatic or intestinal first-pass metabolism. However, it could be worthwhile to measure the other pathways of elimination, such as the composition of the exhaled air after oral administration, in order to reveal the unknown 78% (See Table 1) of the oral dose (only 22% is known as mentioned above), according to the authors [89].

## 4. In Vivo Biological Activity

### 4.1. Anti-Cancer Activity

Pine (genus *Pinus* and family Pinaceae) needle (leaves) oil containing α-pinene is applied as an anti-cancer agent in Traditional Chinese Medicine. For example, Chen, Liu, Li, Mao, Zhang, Huang, Jin and Ye [36] investigated the ability of α-pinene to suppress hepatoma carcinoma BEL-7402 cells in vitro and in vivo. Under in vitro conditions, BEL-7402 cells were treated with α-pinene at doses ranging from 0.125 to 8 mg/L, and assessed using MTT method at 24, 48, and 72 h. α-pinene was found to display an in vitro inhibitory rate of 79.3% (*p* < 0.05) at 72 h on BEL-7402 cell growth, being both dose- and time-dependent. Mice having BEL-7402 xenograft tumor cells treated with α-pinene showed significant suppression over 5-FU (fluorouracil). Tumor growth suppression due to cell death, slow-growing cells, and lower weight were observed when compared to control. The study also showed a decrease in Cyclin B protein levels after α-pinene administration, which is associated with G2 phase transition to M phase cell cycle arrest. Furthermore, the authors found that Chk1 and Chk2 levels were upregulated, while CDC25 and CDNK1 levels were downregulated as a result of α-pinene treatment. Chk1 and Chk2, which is the core of DNA injury check, suppress CDC2 activity and block mitosis by inhibiting CDC25 phosphatase activity through phosphorylation. Therefore, they presumed that α-pinene-induced cell cycle arrest is regulated by Chk2 and CDC25C, thereby effectively inhibiting cell growth [36].

In the work carried out by Matsuo, Figueiredo, Arruda, Pereira, Scutti, Massaoka, Travassos, Sartorelli and Lago [33], they aimed to study the α-pinene-induced cell death mechanism and its medical use in treating malignant melanoma. In the experiment, in vivo melanoma B16F10Nex2-injected mice were treated with *α*-pinene intraperitoneal doses of 100 µL at 10 mg/mL on alternate days. After 12 days, mice lungs were collected and tested for metastatic nodules. The results showed that α-pinene was able to induce apoptosis, shown by early disruption of mitochondrial function, ROS production, increase in caspase-3 activity, heterochromatin aggregation, DNA fragmentation, and exposure of phosphatidyl serine on cell surface [33]. Notably, α-pinene has been shown to be effective in experimental metastatic melanoma healing, reducing the number of lung cancer nodules. This was the case in the first report on apoptotic and anti-metastatic effect using α-pinene [33].

In another study, β-pinene demonstrated higher antiproliferative effect against A-549 (IC_50_ of 85.0 µM) and weak activity against DLD-1 cell lines, in a study conducted using *Nigella sativa* L. [97]. As per the literature, β-pinene possesses moderate antiproliferative activity against human erythroleukemic K562 cells [98]. In another example, the main constituents of *Salvia officinalis* (sage) EO, composed of thujone, β-pinene, and 1,8-cineol, was revealed to be cytotoxic on cell line of the oral cavity (UMSCC1). Beyond the concentration of the IC_50_ of 135 µg/mL, sage EO decreased UMSSC1 cell viability to a minimum, while low concentrations of EO increased cell viability [99].

### 4.2. Anti-Allergic Activity

The therapeutic effects and underling mechanisms of α-pinene in ovalbumin (OVA)-sensitized allergic rhinitis (AR) model were studied by Nam et al. [69]. In this study, OVA-sensitized mice were pre-treated with *α*-pinene orally for 10 days before and after intranasal OVA challenge. A significant reduction in clinical symptoms of AR was found, such as nasal, eye, and ear rubs; spleen weight reduction; and production of inflammatory cytokines levels such as tumor necrosis factor-α (TFN-α) and interleukin-6 (IL-6) in OVA-sensitized mice. A marked decrease in interleukin (IL)-4 levels in spleen tissues, nasal immunoglobulin E, TFN-α, intercellular adhesion molecule-1, and macrophage inflammatory protein-2 levels in nasal mucosa were also stated. After oral administration of α-pinene, human mast cell activation was inhibited and, consequently, the activity of receptor-interacting protein 2 (RIP2), IkB kinase (IKK)-β, and nuclear factor kappa-B kinase (NF-kB) was increased [69,100], and caspase-1 was inhibited. Taken together, the authors suggested that α-pinene is a promising anti-allergic compound. In contrast, Myoga (*Zingiber Myiga Roscoe*), also called Japanese ginger, containing both α- and β-pinene, has been suggested as a skin irritant, according to Wei, et al. [101]. The authors also included α-, β-pinene, and R-(+)-limonene in acute dermal irritation assays on guinea pigs and found a positive response causing erythema on abdominal skin at concentrations of 4%, 4%, and 20%, respectively.

### 4.3. Anti-Inflammatory Activity 

α-pinene is capable of modulating inflammation. Bae, Park, Choi, Jo, Choi, Hong, Song, Song and Park [64] examined the effects of α-pinene on acute pancreatitis (AP), which is a complicated inflammatory disorder with unknown underlying pathogenesis. AP commences with neutrophils migration and proceeds to pancreatic acinar cell damage [102], then hemorrhagic necrotizing pancreatitis, and finally it results in multiple organ failure. As a result, pancreatic weight (PW) gain is evident due to edema, which is measured as increase of pancreatic weight (PW)/body weight (BW). α-pinene weakens AP severity by inhibiting tissue injury, digestive enzyme production, and pro-inflammatory cytokine production. 18 h-fasted mice were induced for AP via intraperitoneal injection of stable cholecystokinin analogue cerulein (50 µg/kg). One h before the first cerulein injection, a pre-treatment of *α*-pinene was given intraperitoneally (5–50 mg/kg). Mice were sacrificed 6 h after the final cerulein injection for data collection. Reduction of PW/BW ratio and of serum levels of amylase and lipase was evident due to *α*-pinene. Histological damage and myeloperoxidase activity in pancreas and lungs were also reduced as a result of *α*-pinene treatment. Furthermore, *α*-pinene pre-treatment was found to influence the reduction of pancreatic TNF-α, IL-1β, and IL-6 production during cerulein-induced AP followed by cell death and cytokine production in isolated cerulein-treated pancreatic acinar cells in vitro [64].

According to Kapsaski-Kanelli, et al. [103], 12 EO from citrus species were tested and limonene (97.79–56.56% variation among 12 spp studied), β-pinene (8.7–19.16%), γ-terpinene, myrcene, α-pinene (1.74–5.08%), neral, and citral were identified. However, this study did not provide any relationships between the phytochemical content and larvicidal pattern. Previous studies [104] have shown that some commonly found compounds, such as γ-terpinene (most toxic to larvae), α-pinene, 3-carene, (R)-(+)-limonene, myrcene, and terpinen-4-ol, display larvicidal activity. In comparison to other citrus-based EOs, repellent bioassays using S-(−) limonene, citral, and (+)-β-pinene in lemon EO were found to be more effective against adult mosquitoes [104]. It has been revealed that limonene and *β*-pinene activity are both optical isomer forms of *β*-pinene that have been found to be more toxic than *α*-pinene on mosquito larvae, as mentioned by Vourlioti-Arapi, et al. [105]. The active isomers of S-(−)-limonene and (+)-*β*-pinene in lemon EO revealed higher protection of human skin against *A. albopictus* [104].

### 4.4. Antimicrobial Activity

α-pinene is an antimicrobial compound that has been effective against some food-borne pathogens, such as MRSA, *B. cereus*, *E. coli*, and *Campylobacter jejuni* [106], Chen, et al. [107] reported that tea tree oil contains as major components terpinen-4-ol and α-terpineol and α-pinene, and all were found to be active against *S. aureus, S. edipermidis*, and *Propionibacterium acnes* (pathogen involved in acne). The minimum inhibitory concentration (MIC) values of active terpenoids increased in the order α-terpineol, terpinen-4-ol, and α-pinene for all three micro-organisms [108].

In *Sideritis erythrantha* var. erythrantha and *Sideritis erythrantha* var. cedretorum, α-pinene has been identified as the major component of EO (27–28%). EO of both varieties showed antibiotic effects on vancomycin-resistant *Enterococcus faecalis* and ampicillin-resistant *Haemophilus influenzae*. Apart from that, *Sideritis erythrantha* var. cedretorum EO was found to be further effective against MRSA and vancomycin-sensitive *E. faecalis* [87]. da Silva et al. [5] in their study found that only the positive enantiomers of α- and β-pinene reveal antimicrobial activity (MICs ranging from 117 to 4150 µg/mL) against *C. albicans* (killing 100% of inoculum within 60 min) and MRSA (within 6 h). The authors reported that 250 µg/mL of (+)-*α*-pinene and (+)-*β*-pinene decreased cell viability of murine macrophages to 66.8% and 57.7%, respectively.

In South Asian countries like Laos, *Amomum* spp. have been used to treat anemia (dizziness, headache), puerperal fever, postpartum secondary haemorrhage, perineal healing, and uterus retraction. *A. villosum* Lour. and *A. microcarpum* (Zingiberaceae) have been used for steam bath for postpartum recovery. *A. villosum* samples displayed large variation on terpenes composition, with β-pinene (5% as the major constituent in rhizome), camphor, bornyl acetate, terpinen-4-ol, fenchone, D-limonene, and α-terpinene as main constituents. β-pinene displayed antimicrobial effects, possibly accentuated through synergistic activity with other terpene compounds, like 1,8-cineol (eucalyptol) [109], borneol, or linalool.

## 5. α-Pinene and β-Pinene in Clinical Studies

α- and β-pinene are the most widely found monoterpenoids, and can be found in EO of many plant species, such as conifers, e.g., *Pinus* sp. (pine), many *Salvia* sp. (sage), *Rosmarinus officinalis* (rosemary), *Helichrysium* sp., *Pistacia vera* (pistachio), black plum, lesser galangal, *Cannabis sativa*, etc. [110,111]. Nevertheless, only a limited number of clinical trials are available up to date on EO, which contains α- and β-pinene in considerable amounts and indicates these monoterpenes as active constituents. A very early clinical study with *α*-pinene was conducted using a capsule preparation called Gelomyrtol forte (one capsule = 0.3 g myrtol, standardized to at least 20 mg of α-pinene, 75 mg of limonene, and 75 mg of 1,8-cineole) in patients with chronic pulmonary obstruction [112]. The trial with a 7-day duration of therapy with 3 × 30 mg ambroxol (reference) or 4 × 1 capsule of gelomyrtol forte led to a marked amendment in mucociliar clearance. In a latter placebo-controlled, double-blind, and parallel-grouped clinical study including 676 male and female outpatients (aged > or = 18 years) with acute bronchitis undertaken by another research group [113], the same preparation was revealed to be tolerated very well after two weeks of treatment. Furthermore, effect of preparation was found to be superior to those of cefuroxime and ambroxol, based on several ancillary parameters, so authors concluded that it could be a very effective option to treat acute bronchitis.

EO have shown to possess powerful insecticidal effects [114]. However, only a small number of clinical studies referring to α- and β-pinene are available on this subject. For instance, the EO of *Eucalyptus globulus* and *Cinnamomum zeylanicum*, which contain α- and *β*-pinene as the major components, were tested in a single-blind clinical study consisting of 95 cases infested with head louse (*Pediculus humanus capitis*) as compared to permethrin as reference drug [115]. The most effective one was found to be *E. globulus* EO-containing shampoo (*p* < 0.06), while *C. zeylanicum* showed equal efficacy to that of permethrin (*p* = 0.139). On the other hand, the difference was non-significant between *E. globulus*-permethrin groups and *C. zeylanicum*-permethrin groups (*p* > 0.28). The data obtained revealed that *E. globulus* in combination with permethrin or itself could be a beneficial solution for head lice.

Relatively more clinical studies have been found in scientific literature considering *α*- and β-pinene-containing plants. In a very recent review, potential activity of EO was scrutinized against Alzheimer’s disease (AD) related to cholinergic hypothesis, amyloid hypothesis, oxidative stress, and other mechanisms searching well-known scientific databases including PubMed, ScienceDirect, Scopus, and Google Scholar from 1998 to 2018 [116]. In that review, *Salvia* species (sage) with over 900 species from all over the world, was examined to determine its effect on AD. As some *Salvia* species are known to be used for memory enhancement in traditional medicine in Europe and some other countries, their EO, rich in α-and β-pinene, were tested in several clinical studies. The triggering point for those clinical studies was that several *Salvia* species (Lamiaceae) have been found to inhibit cholinesterase (ChE) (acetylcholinesterase and butyrylcholinesterase) in vitro and in vivo—the key enzyme in AD pathogenesis. Perry et al. designed an open label clinical study including 11 patients aged 76–95 years diagnosed with mild -to-moderate AD [117]. Patients received capsules containing 50 µL EO of *S. lavandulaefolia* in sunflower oil. After 6 weeks use of this EO, a statistically significant decrease in neuropsychiatric symptoms and an enhancement in attention were recorded. In a similar clinical study, Tildesley et al. also tested the EO of *S. lavandulaefolia* Vahl. (Spanish sage) given as capsulated in sunflower oil to healthy volunteers (24 adults, mean age: 23 years) in two groups in a placebo-controlled, double-blind, and crossover study [118]. The first group consisted of 20 healthy adults (mean age: 20 years) who received the EO at doses of 50, 100, and 150 µL, while the second group included 24 healthy adults (mean age: 23 years) having EO at 25 and 50 µL. Data indicated that the EO of *S. lavandulaefolia* was effective at 50 µL in both groups, which led to a marked improvement in mood and cognition. EO composition of this species consisted of α-pinene (6.5%), camphene (6.3%), β-pinene (5.4%), myrlene (1.9%), limonene (1.2%), 1,8-cineole (25.8%), and camphor (24.4%) as major terpenoids. Consistently, the plant has also been reported to contain 1,8-cineole and *α*-pinene as main substances in other studies [119], which were linked to the anti-Alzheimer activity of plant through inhibition of ChE enzymes activity, triggered by both synergistic or antagonistic interactions between camphor, 1,8-cineole, and α- and β-pinene [120,121,122,123,124]. In particular, the EO of *S. officinalis* and *S. lavandulaefolia* have been noted to show high efficacy to inhibit these enzymes, leading to the conclusion that the high ChE inhibitory effect of these EO comes from synergistic and antagonistic interactions among the major monoterpenes (camphor, 1,8-cineole, borneol, α- and β-pinene) [124]. Later, *S. lavandulaefolia* EO was revealed to be significantly effective in a pilot open-label study consisting of patients with mild-to-moderate level of AD [123]. However, it was found to be unsuccessful in another clinical study, which may due to several reasons, such as lack of standardization, etc. [125].

On the other hand, the EO composition of *S. lavandulaefolia* and *S. officinalis* L. is known to be similar to some extent, except for their thujone content, while that of *S. officinalis* usually possesses higher content of thujone, which is toxic [126,127,128]. Accordingly, in a clinical study, the EO of *S. lavandulaefolia* and *S. officinalis* were comparatively investigated in a single-blind randomized controlled trial with healthy volunteers (135 healthy adults, mean age: 22 years) [125]. The scores based on Bond–Lader mood scales and cognitive drug research system indicated that the group treated with EO of *S. officinalis* displayed a better outcome for mood and memory performance, compared to the control group. Kennedy et al. also showed the positive action of *S. lavandulaefolia* EO (50 mL) prepared in soft gel with olive oil in a double-blind, placebo-controlled clinical trial including 36 healthy adults with a mean age of 24 years. The results obtained pointed to the fact that *S. lavandulaefolia* EO enhanced secondary memory performance along with attention, whereas it led to a decrease in mental fatigue and an increase in alertness, which were observed to be more obvious 4-h post-dose [129].

## 6. Conclusions

This review shows that pinenes comprise important compounds derived from plants EO that exhibit various biological activities, which makes them useful for various applications and uses, e.g., as fungicidal, flavors, fragrances, antiviral, and antimicrobial agents. Nonetheless, compounds related to α-pinene and β-pinene bioavailability last for a short duration at low concentrations, being rapidly metabolized and eliminated from the body due to their volatile nature. To date, most of the investigations have not studied the bioavailability of *α*-pinene and β-pinene in the human body, though it is clear that these terpenes have antimicrobial, anticancer, anti-inflammatory, and antiallergic properties. Although several in vivo, and more recently, few clinical studies have assessed the pinenes biological effects, further efforts are needed to deepen knowledge in this field.

## Figures and Tables

**Table 1:** Antimicrobial activity of α-pinene and β-pinene.

S. No.	Source/Species	Model	Plant Portion	Method	Result	Ref
**α-pinene**						
1	Sigma Aldrich	*Campylobacter jejuni*	-	Broth microdilution and ethidium bromide deposition	Modulation of antibiotic resistance, by reducing MIC value of ciprofloxacin, erythromycin, and triclosan, up to 512 times. α-pinene also affected antimicrobial efflux systems	[21]
2	-	*Nocardia* sp. Strain (P18.3), *Pseudomonas putida* PX1 (NCIB 10684), *Pseudomonas* sp. strain PIN18 (NCIB 10687), and *P. fluorescens* NCIB 11671	-	Strains were cultured into agar slants with α-pinene (3 g/L in media), and strains growth was recorded	*Nocardia* sp. growth (P18.3) was not remarkable; *Pseudomonas* strains (NCIB 10684, 10687, and 11,671 and PL) increased promptly when α-pinene (0.3%, *v*/*v*) was added	[22]
24	Citrus species	*Propionibacterium acnes*, *Staphylococcus epidermidis*	Peel EO	EO was isolated by hydrodistillation	EO demonstrated outstanding antibacterial properties against *P. acnes* and *S. epidermidis*	[55]
26	Sigma-Aldrich	*Escherichia coli*, *Micrococcus luteus*, *Staphylococcus aureus*, and *Candida albicans*	-	Bioautographic method MIC was measured	(+)-α-pinene exhibited modest activity. (−)-α-pinene was unable to display any activity. α-pinene and β-lactams revealed the highest effects. Although (−)-α-pinene revealed no positive activity, the derivatives like β-lactam, amino ester, and amino alcohol exhibited antimicrobial effects	[56]
28	*Bursera morelensis*	*Candida albicans strains (ATCC 14065, ATCC 32354, donated strain, and CDBB-L-1003)*	Stems (EO)	EO was extracted by hydrodistillation, and GC-MS was used to isolate compounds Disc diffusion and survival curve assay were used	Maximum antifungal activity was attributed to the EO and its constituent, namely, α-pinene. Minimum fungicidal concentration of EO was found to be 2 mg/mL. A slight reduction in *C. albicans* population was recorded after 12 h	[58]
30	-	*Staphylococcus aureus* and *Escherichia coli*	-	Disc diffusion test, broth microdilution, and bacterial death kinetics	Inhibition halos of 11 and 12 mm for gram-positive and -negative strains were obtained at 160 µL/mL, respectively. At 1.25 and 2.5 µL/mL, (+)-α-pinene was able to eliminate bacterial colonies formation at one time of exposure of 2 h for *E. coli* strain	[60]
31	*Syzygium cumini*	*Swiss mice*	Leaves (EO)	MTT assay Cytotoxic effect on macrophages was determined; cells were exposed to α-pinene and tested against Leishmania	Cytotoxic effect of α-pinene against promastigotes of *Leishmania amazonensis* was observed with different cell death percentages (93.7, 83.2, and 58.4%) at different concentrations (100, 50, and 25 mg/mL respectively)	[61]
40	-	*House fly (Musca domestica)*	-	Y-tube and house flies were selected for this test	Solution with lowest concentration did not show significant differences in Y-tube arm choice. (1S)-(-)-α-pinene had maximum repellent efficiency for house flies when compared to (1R)-(+)-α-pinene	[84]
45	Plectranthus barbatus	*Malaria (Anophel es subpictus), dengue (Aedes albopictus), and Japanese encephalitis (Culex tritaeniorhynchus) mosquito vectors*	EO (leaves)	GC and GC--MS were performed; larvicidal activity of EO (40, 80, 120, 160, and 200 µg/mL) and its constituents eugenol, α-pinene, and β-caryophyllene (12–100 µg/mL each) were determined by WHO methods. Mortality of larvae was measured at 24 h after exposure	EO showed substantial larvicidal effects with LC_50_ values of 84.20, 87.25, and 94.34 µg/mL for the selected mosquito species. For *Anapheles subpictus*, eugenol, α-pinene, and β-caryophyllene revealed larvicidal effects (LC_50_ = 25.45, 32.09, and 41.66 μg/mL), followed by *Aedes albopictus* (LC_50_ = 28.14, 34.09, and 44.77 μg/mL) and *Culex tritaenior hynchus* (LC_50_ = 30.80, 36.75, and 48.17 μg/mL, respectively)	[83]
**β-pinene derivatives**						
27	-	*Klebsiella pneumoniae, Enterobacter aerogenes, S. aureus, S. epidermidis, and Candida albicans*	-	25 3-cyanopyridine compounds of β-pinene were prepared; MIC value was recorded using serial two-fold dilution method	MICs values of all derivatives ranged from 15.6 to 125 mg/l	[57]
29	-	*Candida* spp.	-	MIC and MFC values and microbial death curve after treatment with (+)-β-pinene enantiomers	MIC values ranged from <56.25–1800 µmol/L (+)-β-pinene. After ergosterol addition, MIC value of (+)-β-pinene was not altered, but was altered with sorbitol addition. (+)-β-pinene displayed anti-biofilm activity against multiple *Candida* species	[59]
**α- and β-pinene**						
22	Dep. Pharmaceutical Sciences, Ponta Grossa, Brazil	*Gram-positive bacteria (Staphylococcus aureus, S. epidermidis, S. pneumoniae, and S. pyogenes)*	-	MIC value, viable cells count	All studied bacterial strains were sensitive to α- and β-pinene. MIC values ranged from 5 (α-pinene x *S. epidermidis* SSI 1; ATCC 12228; *S. pyogenes* ATCC 19,615; and *S. pneumoniae*) to 40 μL/mL (β-pinene x *S. epidermidis* ATCC 12228). Few bacterial strains were resistant antibiotics, mainly gentamicin. *S. aureus* was resistant to α- and β-pinene	[53]
23	Sigma-Aldrich	*Antimicrobial: Escherichia coli (ATCC 11775, Staphylococcus aureus (ATCC 25923), Bacillus cereus (ATCC 11778), and Candida albicans (ATCC 10231).* *Antimalarial: Plasmodium falciparum (FCR-3)*	-	Disc diffusion method. MIC was investigated. Antimalarial properties were analyzed using the tritiated hypoxanthine incorporation assay	(+)-β-pinene was approximately two to 12 times more effective as compared to (+)-α-pinene against both gram-positive and negative bacteria, as well as *C. albicans*. (+)-α-pinene shows 250-fold more antimalarial activity than (+)-β-pinene	[54]
25	Sigma-Aldrich	*Candida albicans, Cryptococcus neoformans,* *Rhizopus oryzae, and methicillin-resistant Staphylococcus aureus (MRSA)*	-	Two-fold serial dilution method was used to evaluate MIC for all the strains	MIC values of α- and β-pinene enantiomers were found to be from 117 to 6250 µg/mL. *C. albicans* exhibited higher sensitivity to α- and β-pinene enantiomers than MRSA. Positive enantiomers possess capability to kill 100% of *C. albicans* in 60 min., and 6 h was required for total killing of MRSA	[5]

**Table 2:** Anticoagulative/antiplatelet and anti-inflammatory activity of α-pinene and β-pinene.

S. No.	Compound	Source/Species	Model	Plant Portion	Method	Result	Ref
**Anticoagulative/Antiplatelet**							
3	α-pinene derivatives (6β,9-dihydroxy-(+)-α-pinene and 9-hydroxy-(+)-α-pinene-6β-O-D-glucoside)	*Angelica sinensis* (Oliv.) Diels	New Zealand white rabbits	Aerial parts	Two α-pinene derivatives were extracted from aerial parts (10 g). Thrombin time and platelet aggregation methods were used to establish the anticoagulative properties in vitro	Isolated α-pinene derivatives slightly prolonged thrombin time and strongly prevented platelet aggregation. This effect seems to be due to prevention of thromboxane A_2_ synthesis or agitation of Ca^2+^ in platelet	[24]
**Anti-Inflammatory**							
41	α-pinene enantiomers	*Juniperus oxycedrus*	Human chondrocyte	EO	Chondrocytes were cultured and exposed to noncytotoxic doses of α-pinene enantiomers	(+)-α-pinene (1) shows maximum suppression of IL-1β-induced inflammatory and catabolic pathways	[74]
42	α-pinene	-	Male C57BL/6 mice (peritoneal macrophages)	-	Cytotoxicity was determined by MTT method. Cytokine assays were executed for IL-6 and TNF-α by following modified ELISA method. Western blotting was used to analyze protein expression	Up to 20 µL, α-pinene was not cytotoxic. α-pinene reduced nitrite oxide, and IL-6 and TNF-α formation, in macrophages of rats. MAPK/NF-kB pathway activation plays an essential role in inflammatory activities. α-pinene exhibited inhibitory activity on NF-kB activation	[73]
43	α-pinene	*Frankincense oil (Boswellia carterii)*	Kunming mice	-	Frankincense oil was extracted from Boswellia carterii, and three compounds, namely, α-pinene, linalool, and 1-octanol, were isolated using GC-MS. Frankincense oil, water extracts, and their constituents were screened against xylene-stimulated edema and formalin-sensitized hind paw edema in rat model for determining the anti-inflammatory and anti-analgesic properties.	Frankincense oil possesses higher anti-inflammatory and anti-analgesic effects than rats administered with water extract. Mixtures of the three constituents have higher pharmacological properties on hind-paw inflammation and COX-2 over expression than used individually	[75]
46	α-pinene	*Sigma-Aldrich*	Wood rats (Neotoma species)		Selected wood rats were sacrificed and intestine removed rapidly from stomach	α-pinene is not a PgP substrate	[77]
47	α-pinene		Wistar rats	-	Selected rats were cannulated via their lateral ventricles for capsaicin administration (100 µg). α-pinene at various concentrations (0.1, 0.2, and 0.4 µM) was administered	0.2 and 0.4 μM concentrations of α-pinene were able to decrease nociception. A marked increase in COX-2 expression in capsaicin-treated rats was observed, which was prohibited by 0.4 μM α-pinene	[78]

**Table 3:** Anti-tumor activity of α-pinene and β-pinene.

S. No.	Source/Species	Model	Plant Portion	Method	Result	Ref.
**α-pinene**						
4	*Schinus terebinthifolius*	Male C57BL/6 mice and B16F10 murine melanoma cell line	Fruits	α-pinene was extracted from ripped fruits and injected into infected mice. Selected cells were cultured and maintained in culture medium	α-pinene-stimulated apoptosis was by early disruption of mitochondrial potential, ROS formation, enhanced caspase-3 activity, heterochromatin deposition, DNA fragmentation, and phosphatidylserine exposure on cell surface	[33]
5	-	C57/BL6 mice	-	α-pinene under aesthetic chamber and mouse cage, and in vitro effects	No effect was found on melanoma cell proliferation in mice under in vitro use of α-pinene	[34]
6	*Pinus massoniana*	Hepatoma carcinoma BEL-7402 cells	Needles	Selected cells were cultured and maintained in RPMI-1640 medium. Cell viability was checked by MTT assay. Cell cycle arrest was observed by flow cytometry. Western blotting was performed to know protein expression	α-pinene prevented BEL-7402 cells by arresting cell growth at G2/M, down regulating Cdc25C mRNA and protein expression, and decreasing cycle dependence on kinase 1(CDK1) action	[35]
7	*Pinus massoniana*	Hepatoma carcinoma BEL-7402 cells	-	α-pinene was isolated from pine needles. Selected cells were cultured and maintained in RPMI-1640 medium. MTT and flow cytometry assays were used for determination of cytotoxicity and cell cycle regulation, respectively.	Liver cancer cell growth was prevented in vitro and in vivo (respectively, 79% and 69.1% inhibitory rate); Chk1 and Chk2 levels were up-regulated; and Cyclin B, CDC25 and CDK1 levels were down-regulated	[36]
9	Pine	Human hepatocellular carcinoma cells (HepG2 cell)	Pine needle	HepG2 cell was administered with α-pinene and cell cycle alteration was analyzed by flow cytometry	α-pinene prevented HepG2 cells proliferation dose-dependently. α-pinene arrested HepG2 cells at G_2_/M phase. miR-221 expression was down-regulated in HepG2 cell treated with α-pinene	[37]
10	-	HepG2, MCF-7, A549, and PC-12 cancer cell lines	-	Cell viability was determined by MTT assay, apoptosis and cell cycle analyses were conducted using flow cytometry	α-pinene inhibited miR221 expression, leading to G2/M-phase cell cycle arrest and activation of CDKN1B/p27-CDK1 and ATM-p53-Chk2 pathways that suppress human hepatoma tumor progression	[38]
11	-	Mouse xenograft model		Cytotoxicity was analyzed using MTT assay, and apoptosis and cell cycle study were performed *in vitro* by flow cytometry	α-Pinene prevented human prostate cancer cell growth and stimulated apoptosis and cell cycle arrest in the cell line-based model. α-Pinene administration stimulated apoptosis in xenograft tumors as measured by TUNEL	[39]
13	Sigma-Aldrich	Chinese hamster (V79-Cl3) cell line	-	Cells (3 × 10^5^ per dish) were exposed at varying doses of α-pinene (0, 25, 30, 35, 40, and 50 µM) for 1 h	Cells morphological analysis revealed a significant enhancement in cell. Apoptotic cells were found at 40 and 50 µM. Genetic instability was stimulated by α-pinene, interfering in mitotic process and causing irregularity in 50% of cells. α-pinene stimulated oxidative stress and led to DNA damage	[41]
***β*-pinene-based thiazole derivative**						
12	-	Human cervical carcinoma HeLa cells, colon cancer CT-26, and human hepatocarcinoma SMMC-7721 cell lines	-	Mechanism of compound 5 g (β-pinene-based thiazole derivatives) on cytotoxicity, DAPI, Annexin-V/PI, JC-1, DCFDA staining, and Western blot assay were performed	Studied compound prevented HeLa cells proliferation through apoptosis stimulation and cell cycle arrest at G0/G1 phase, dose-dependently. Studied compound increased ROS level; caused a reduction in mitochondrial membrane potential; enhanced mitochondrial cytochrome *C* discharge; and impacted Bax, Bcl-2, caspase-3, and caspase-9 expression	[40]
**α- and β-pinene**						
8	-	A-549 and H 460 cancer cell line	-	Selected cells were maintained in RMPI-1640 medium. MTT assay was used to analize cell viability. Cell cycle regulation was checked by flow cytometry	A significant inhibitory effect of the mixture of paclitaxel (PAC) with α-pinene or β-pinene was recorded on non-small-cell lung cancer cell lines	[27]

**Table 4:** Preclinical antioxidant activity of α-pinene and β-pinene obtained from different sources.

S. No.	Source/Species	Model	Plant Portion	Method	Result	Ref
**α-pinene**						
14	Sigma Aldrich	Cultured human blood cells	-	Varying doses of α-pinene (at 0, 10, 25, 50, 75, 100, 150, and 200 mg/L doses) were administered in human blood cells for 24 and 48 h. Cytotoxicity was evaluated using LDH and MTT methods. DNA damage was detected using micronucleus assay, chromosomal aberration, and 8-oxo-2-deoxyguanosine (8-OH-dG). Total antioxidant capacity (TAC) and total oxidative stress (TOS) were measured.	Reduced cell viability was recorded by α-pinene (200 mg/L) administration. No changes were detected in the rates of genotoxicity endpoints. Dose-dependent changes were recorded in TAC and TOS levels. TAC levels were enhanced after supplementation with α-pinene (25 and 50 mg/L), while TOS level were reduced only at 200 mg/L of α-pinene on human lymphocytes	[42]
32	Aldrich chemicals	Sprague–Dawley rats	-	Pinene dissolved in 10% ethanol and 90% corn oil at 40 mg/kg b.w. was injected three times into healthy mice with 180–200 g weight. Comparative assessments of these mice were performed with few other mice administered with phenobarbital (0.9% NaCl). Control mice received a vehicle (10% ethanol and 90% corn oil)	No visible alterations were recorded in liver microsomal membrane proteins of mice after administration of the different terpenoids. No effect was found in the amount of cytochrome present in mice liver. Terpenoids administered mice had remarkable stimulation on PB P-450	[62]
34	Sigma chemicals	Rat small intestine epithelial (IEC-6) cells	-	DPPH assay was examined at varying doses of α-pinene (25, 50, 100, 200, 300, and 400 µg/mL). IEC-6 cells were exposed in 10 mM aspirin (A) with and without α-pinene for 24 h. SOD, mitochondrial SOD, and glutathione activities were assessed	With enhancing doses of α-pinene until a maximum dose (400 µg/mL) was reached, the anti-DPPH activity was found to increase. FRAP activity was enhanced by increasing the dose of α-pinene (up to 300 µg/mL). Lower dose of α-pinene was unable to display any effect on cell viability. Exposure of aspirin with α-pinene displayed an expansion in cytotoxicity, compared to exposure of aspirin alone. Aspirin caused a negative alteration in cell morphology; however, exposure to aspirin with α-pinene did not lead to morphological changes	[66]
35	-	Human skin epidermal keratinocytes (HaCat cells)	-	HaCat cells were kept in DMEM administration and then divided into four groups, i.e., non-irradiated control cells, α-pinene (30 µm)-treated cells, UVA (10 J/cm^2^)-irradiated cells, and α-pinene-pretreated (30 min before) and UVA-irradiated cells. Cellular damage was caused by the stimulation of UVA-irradiation (10 J/cm^2^)	Up to 30 µm α-pinene, no cell death was observed. Cell viability decreased significantly after UVA exposure. UVA-stimulated cytotoxicity was inhibited by α-pinene pretreatment. UVA irradiation enhanced ROS formation. However, α-pinene pretreatment significantly inhibited ROS formation. UVA-exposed cells exhibited higher peroxidation levels, decreased by α-pinene	[67]
36	Sigma chemicals	Swiss Albino mice	-	Cell damages was triggered by UVA-irradiation (10 J/cm^2^ per day) for 10 days. Before-exposure rats were administered with α-pinene (100 mg kg/b.wt). Antioxidant enzymes and oxidative stress were analyzed. In the rat skin, histopathological analysis was also carried out	UVA exposure decreased the level of SOD, CAT, GPx, and GSH in mouse skin, and increased ROS formation. Peroxidation level was higher in UVA-exposed rat, compared to non-irradiated control and α-pinene-alone-administered mice. α-pinene administration before UVA-exposure significantly enhanced SOD, CAT, GPx, and GSH activities, and significantly decreased the level of lipid peroxidation. α-pinene-treated mice exhibited greater iNOS and VEGF expression than non-treated control rats	[68]
**β-pinene**						
52	Sigma-Aldrich	*In vitro*		DPPH, ABTS, and FRAP assays	IC_50_ values for DPPH and ABTS were 3116.3 μg/mL and 2245.0 μg/mL, respectively. FRAP value was 6.5 μM Fe/mg pinene.	[85]

**Table 5:** Gastroprotective activity of α-pinene and β-pinene.

S. No.	Source/Species	Model	Plant Portion	Method	Result	Ref
**α-pinene**						
16	Sigma-Aldrich and *Hyptis* species	Swiss mice	EO	Different doses of ethanol and indomethacin (10, 30, and 100 mg/kg) were used to induced gastric ulcers. Acute gastric lesions were introduced into rats, and these rats fasted for 12 h. After that, rats were administered with 0.5 mL of vehicle (0.1% tween-80), ranitidine (40 mg/kg), and α-pinene (10, 30, and 100 mg/kg) dissolved in vehicle	α-pinene decreased ethanol-induced gastric mucosa lesion and produced gastroprotective effects similar to ranitidine (40 mg/kg). There were no remarkable variations between lesions area of α-pinene and vehicle-pretreated mice	[46]
17	*Pistacia atlantica*	Wistar strain mal albino rats	Oleoresin (EO)	EO was supplemented with varying doses in the selected mice. Mice were kept under observation after 72 h to determine toxicity (restlessness, dullness, and agitation). 80% ethanol was supplemented. Rats were sacrificed 2 h after to remove stomachs. Gastric ulcers were determined using microscopy. *H. pylori* strains were cultured	EO was harmless up to 2000 mg/kg. Strains of *H. pylori* were sensitive to EO. MIC values ranged from 0.275 to 1.100 mg/mL. EO considerably decreased ethanol-stimulated peptic ulcer. Pretreatment with EO reduced ethanol-stimulated gastric tissue damage and necrosis	[47]
19	Sigma-Aldrich	C57BL/6 mice	-	Mice were fasted for 18 h, followed by stimulation of acute pancreatitis (AP). AP was treated in every h (for 6 h) by cerulein (50 µg/kg i.p.). α-pinene was vaccinated at varying doses before the first cerulein injection.	After α-pinene stimulation, PW/BW proportion was reduced. Lipase and amylase levels were enhanced in serum during cerulein-induced AP, whereas α-pinene decreased them	[64]
33	Aldrich cehmicals	Barred Rock Chickens	-	Livers of 16- to 18-day-old embryos of identified chickens were cultured and compared with white Leghorn embryos for knowing the behavior. Porphyrins were analyzed fluorimetrically	α-pinene formed some amount of porphyrins in chick embryo liver cells. α-pinene led to the deposition of 100-150 porphyrins/mg (copro- and protoporphyrins) protein at the highest screened dose (1 mM).	[65]
**α- and β-pinene**						
15	*Eucalyptus tereticornis*	Male Wistar rats	EO of whole plant	Liquid test meal comprising phenol red was supplemented, and gastric emptying was analyzed after varying time intervals	Studied species and their components reduced gastric retention in mice, and α- and β-pinene enhanced gastric tonus in anesthetized rats	[45]

**Table 6:** Neuroprotective activity and other nervous system’ effects of α-pinene and β-pinene.

S. No.	Source/Species	Model	Plant Portion	Method	Result	Ref
**α-pinene**						
18	Santa Cruz Biotechnology Inc. (Dallas, TX, USA)	Mice	-	α-pinene and zolpidem were supplemented orally pre-pentobarbital injection (45 mg/kg)	α-pinene displayed sleep improving activity through a direct binding to GABA_A_-benzodiazepine receptors (GABA_A_-BZD). α-pinene (12.5, 25, 50, and 100 mg/kg) reduced sleep latency and enhanced the duration of NREMS without any action on REMS and delta effects	[48]
19	Tokyo Chemical Industry	Mice	-	Rats were exposed to α-pinene and water as negative control for 60/90 min. Followed by inhalation, quantitative measurement of α-pinene in brain and gene expression was undertaken. EPM test was performed for determining the anxiolytic-like effect in rats	Distance was enhanced (*p* < 0.001, d = 3.4, and 1-β = 0.98) when mice inhaled α-pinene for 60 min. α-pinene dose for 60 min in brain was higher when compared to 90 min. BDNF mRNA expression in olfactory bulb and hippocampus was almost similar at 60 min inhalation than at 90 min. TH mRNA expression in middle brain at 60 min was higher	[49]
20	Sigma-Aldrich	Rat pheochromocytoma cells (PC12)	-	PC12 viability was checked using MTT method. Cells were incubated for 30 min with DCFH-DA. Intracellular ROS formation was measured by DCFH-DA assay	α-pinene pretreatment led to cell viability loss and alteration in cell morphology. α-pinene prevented intracellular ROS production, and increased CAT, SOD, GPx, GR, and HO-1 expression	[50]
21	*Salvia lavandulifolia*	Human astrocytoma 373-MG cell line	Aerial parts	Cytotoxicity was evaluated using MTT method. DCFH-DA method was used to evaluate intracellular ROS formation. TBARS method was used for lipid peroxidation, and spectrometric techniques and Western blot for enzymatic activity and protein expression	Viability of α-pinene-treated cells (10–250 mM) was not reduced. Earlierα-pinene (at 10, 25, 50, and 100 mM dosed) administration enhanced cell viability in U373-MG dose-dependently. (IC_50_ = 79.70 mM). α-pinene pre-treatment preserved U373-MG cells against H_2_O_2_-stimulated oxidative damage and cell morphology, prevented ROS synthesis and lipid peroxidation, and enhanced antioxidant status	[51]
38	Ducrosia anethifolia	Wistar rats	Aerial parts (leaves and flowers)	Rats were administered with the EO of the species (500 mg/kg). Mortality and morbidity were analyzed. Pentylenetetrazole (PTZ, 80 mg/kg) was injected for stimulating convulsions in mice. Administration of rats 30 min before treatment with PTZ, diazepam (2 mg/kg), EO (25, 50, 100, and 200 mg/kg), and α-pinene (0.2 and 0.4 mg/kg) were supplemented. Mice behavior was recorded with a CD camera.	EO exhibited activity against PTZ- stimulated seizures, which can significantly decrease convulsing in rats. Death rate and PTZ-stimulated seizures decreased significantly after pretreatment with EO and α-pinene. EO and α-pinene were able to reduce oxidative stress features significantly after seizures stimulated by PTZ	[70]
44		Wistar rats		Rats were administered with varying doses (0.003%, 0.03%, and 0.3%) of α-pinene odor. Mice were remained in cages at a constant room temperature and were kept at 12 h dark and 12 h light condition with food and water. After being given the odor of varying doses of α-pinene, rats were exposed to different, unfamiliar environments	There was alteration in body temperature (abrupt increase) at 0.03% α-pinene after the transfer from home cage. However, 0.003% and 0.3% α-pinene odor decrease the stress stimulated hyperthermia in mice. 0.003% and 0.03% did not display any alteration in heart rate, but 0.3% led to changes. Varying doses of α-pinene bind to different olfactory receptors and stimulate different type of neuronal activities	[76]
**α- and β-pinene**						
39	Sigma-Aldrich	Male Swiss Albino mice (*Mus musculus*)	-	Rats were treated with α- and β-pinene. Pretreated mice were supplemented with pentylenetetrazole (80 mg/kg i.p.) to induce seizures followed by one h of treatment. Mice were sacrificed by cervical dislocation, and brains, hippocampus, and striatum were removed immediately for neurochemical analysis	Significant seizure intensity reduction was observed at 400 mg/kg. Mixture of 400 mg/kg α- and β-pinene significantly enhanced the latency of the first convulsion. β-pinene and mixture (400 mg/kg) significantly enhanced the mortality time of rats. α-pinene and equimolar mixture remarkably decreases the hippocampal nitrite level and striatal content of dopamine and norepinephrine	[72]

**Table 7:** Effects of α-pinene and β-pinene on nervous system and kidney.

S. No.	Source/Species	Compound	Model	Plant Portion	Method	Result	Ref
**Respiratory system**							
37	-	α-pinene	BALB/c female mouse	-	α-pinene (0.1, 1, and 10 mg/kg) was administered to rats once a day for 10 days, 1 h before or 1 h after intranasal OVA challenge. HMC-1 cells were cultured into IMDM medium. Cell viability was assessed	Pretreatment with α-pinene reduced clinical symptoms, i.e., reduction in number of nasal, eye, and ear rubs and spleen weight; a decline in IL-4 levels; and a reduction in the level of nasal immunoglobulin E in OVA-induced rats	[69]
48	Fluka chemicals	α- and β-pinene enantiomers	OF1 (I.O.P.S. Caw) and KTL [(Hsd/Ola):NIH/(SPF)] male mice	-	Rats were placed in steel cages. Then, rats were kept in glass tubes (body plethysmograph). Rats were exposed (15 min) to selected pinene enantiomers. Differential pressure transducer attached with pneumotachograph was used to analyze inspiratory (VI) and expiratory (VE) air flow	Initially, no irritation was recorded in rats kept in room air. After the introduction of pinene enantiomers, the irritation was recorded, which indicates D-enantiomers were efficient sensory irritants. RD_50_ for pinene D-enantiomers was almost equal	[79]
49	Fluka chemicals	α-pinene enantiomers	BALB/c mice	-	Sensory irritation, airflow limitation, and pulmonary irritation of pinene have been studied	Sensory irritation was observed on the upper respiratory tract by (+) enantiomer during exposures 100 to 369 ppm. Initial dose was 70 ppm, which is nearest to the non-effective level (40 ppm) in humans. 200 ppm and higher concentrations triggered airflow limitations	[80]
50	Sigma-Aldrich	α-pinene	Human volunteers	-	Human volunteers were exposed in an exposure chamber for inhalation (2 h, 50 W) of α-pinene (10–450 mg/m^3^). After the exposure, capillary blood, urine, and exhaled air were determined	Absolute uptake of α-pinene enhanced linearly with exposure dose. α-pinene dose was firstly increased rapidly in arterial blood during the exposure, and then leveled off up to the end of exposure. Some undesirable effects were recorded during the exposure	[40]
**Nephropathy**							
51	-	Piperazine ferulate tablets, + eucalyptol, limonene, and pinene soft capsules	Children with IgA nephropathy	-	Control group patients were administered with conventional or hormone therapy. Observation group patients were supplemented with piperazine ferulate tablets (0.1 g/dose and 3 times/day) coupled with eucalyptol, limonene, and pinene enteric soft capsules (0.1 g/dose and two times/day) for six months.	Effective rate of observational group (12 patients) was remarkably higher than hormone group (18 patients). Variations in serum IgA, fibronectin, and complement C3 of selected two groups were not statistically significant	[82]

**Table 8:** Bioavailability of α-pinene and β-pinene.

	Exposure	Uptake	Distribution	Elimination		
				Exhale Air	Blood	Urine
**Inhalation** (8 volunteers, with light exercise-50W) [81,95,96]						
**α-pinene (+)**	2 h 450, 225, or 10 mg/m^3^	Relative net uptake 59–62% *	t_max_ 120 min c_max_ 20 µMol/L(for 450 mg/m^3^) c_max_ 10 µMol/L(for 225 mg/m^3^) (exposure concentration depended) * c_max_ 10 µMol/L	7.7%	**cl_21h_** 1.9 lkg^−1^h^−1^	0.001% In 30 min 4% of total uptake as cis and trans verbenol
					**t_1/2_** (3 phases α, β, γ) α-4.8 min β-38 min γ-695 min	
**α-pinene (−)**	450 mg/m^3^			7.5%	**cl_21h_** 1.16 lkg^−1^h^−1^	
					**t_1/2_** α-5.6 min β-40 min γ-555 h	
**β-pinene**	450 mg/m^3^ *	Relative net uptake 66% *	**c_max_** 3 µMol/L *	* 5.7%	* cl_21h_ 0.5 lkg^−1^h^−1^ * t_1/2_ α-5.3 min β-41 min γ-25 h	Not available
**Dermal application** in vitro [93], Ex vivo [92]						
**α-pinene**	1000 µL (concentration is not provided) for 27 h	P_app_ 6.49 × 10^−5^ cm/s				
	100 mg/cm^2^ applied on 0.65 cm^2^ at 37 °C ¥		c_max_ 40 µg/cm^2^ t_max_ 15 min in SC			
**β-pinene**	1000 µL (concentration is not provided) for 27 h	P_app_ 4.48 × 10^−5^ cm/s				
	100 mg/cm^2^ applied on 0.65 cm^2^ at 37 °C ¥		c_max_ 290 µg/cm^2^ t_max_ 60 min in SC			
**Oral administration** (four volunteers) [89]						
**α-pinene**	9 mg (66 µmol)		Unmetabolized state—not detected (<4 µg/L)		**t_1/2_** MYR-1.7 h tVER-1.0 h cVER-0.8 h	**t_max_** 1.6 h (metabolites)
			**t_max_** 1–3 h Metabolites			**t_1/2_** MYR-1.5 h cVER and tVER-1.6 h MYRA-1.4 h
			**c_max_** MYR-11 µM tVER-26 µM cVER-9.3 µM			**cl_24h_** MYR-1.5%, cVER-5.6%, tVER-4.1% MYRA-6.7%.
				78% unknown elimination, which could be exhalation or first-pass metabolism		

* Chamber vapour proportions α-pinene-54%, β-pinene-11%, 3-carene-35%; ¥ α-pinene 4.8%, β-pinene 1.1%, eucalyptol 3.3%, camphor 5.7%, and menthol 3.8%; trans-verbenol (tVER), *cis*-verbenol (cVER), myrtenol (MYR), myrtenic acid (MYRA), αPNM3, and αPN-M1, which are metabolites of α-pinene.
